# piRNA-guided slicing of transposon transcripts enforces their transcriptional silencing via specifying the nuclear piRNA repertoire

**DOI:** 10.1101/gad.267252.115

**Published:** 2015-08-15

**Authors:** Kirsten-André Senti, Daniel Jurczak, Ravi Sachidanandam, Julius Brennecke

**Affiliations:** 1 Institute of Molecular Biotechnology of the Austrian Academy of Sciences (IMBA), Vienna Biocenter (VBC), 1030 Vienna, Austria;; 2 Department of Genetics and Genomic Sciences, Mount Sinai School of Medicine, New York, New York 10029, USA

**Keywords:** *Drosophila* oogenesis, Piwi pathway, transposon silencing, piRNA biogenesis

## Abstract

PIWI clade Argonaute proteins silence transposon expression in animal gonads. Their target specificity is defined by bound ∼23- to 30-nucleotide (nt) PIWI-interacting RNAs (piRNAs) that are processed from single-stranded precursor transcripts via two distinct pathways. Primary piRNAs are defined by the endonuclease Zucchini, while biogenesis of secondary piRNAs depends on piRNA-guided transcript cleavage and results in piRNA amplification. Here, we analyze the interdependencies between these piRNA biogenesis pathways in developing *Drosophila* ovaries. We show that secondary piRNA-guided target slicing is the predominant mechanism that specifies transcripts—including those from piRNA clusters—as primary piRNA precursors and defines the spectrum of Piwi-bound piRNAs in germline cells. Post-transcriptional silencing in the cytoplasm therefore enforces nuclear transcriptional target silencing, which ensures the tight suppression of transposons during oogenesis. As target slicing also defines the nuclear piRNA pool during mouse spermatogenesis, our findings uncover an unexpected conceptual similarity between the mouse and fly piRNA pathways.

## Abstract (precis)

In this study, Senti et al investigate how cytoplasmic post-transcriptional silencing influences transcriptional silencing in the nucleus. They show that Piwi-bound piRNA populations depend almost exclusively on prior piRNA-guided transcript slicing, thus providing further insight into the regulation of piRNA biogenesis in the developing *Drosophila* ovary.

The invasion of eukaryotic genomes by transposable elements (TEs) is an ancient genetic conflict that triggered the evolution of efficient defense systems on the host side (Kazazian 2004; Slotkin and Martienssen 2007). In fungi, plants, and animals, small RNA pathways contribute significantly to TE silencing (Malone and Hannon 2009). The major TE repression system in animal gonads is the PIWI-interacting RNA (piRNA pathway). It uses PIWI clade Argonaute proteins loaded with 22- to 30-nucleotide (nt) piRNAs (Malone and Hannon 2009; Iwasaki et al. 2015).

Three core principles characterize the piRNA pathway: First, piRNAs are processed from single-stranded precursor RNAs, which are derived from hundreds of genomic piRNA source loci. The predominant piRNA precursors are long noncoding RNAs (often enriched in TE sequences) and genic mRNAs where piRNA production is largely restricted to 3′ untranslated regions (UTRs) (Senti and Brennecke 2010;

Iwasaki et al. 2015). piRNA source loci that give rise to large populations of piRNAs are called piRNA clusters and act as the heritable TE sequence repositories for the pathway.

Second, two distinct cytoplasmic piRNA biogenesis modes exist. During primary piRNA biogenesis, the endonuclease Zucchini defines 5′ and 3′ ends of piRNAs via consecutive precursor cleavages (Brennecke et al. 2007; Pane et al. 2007; Lau et al. 2009; Malone et al. 2009; Haase et al. 2010; Olivieri et al. 2010; Saito et al. 2010; Ipsaro et al. 2012; Nishimasu et al. 2012; Han et al. 2015; Mohn et al. 2015). Secondary piRNA biogenesis is initiated by piRNA-guided target cleavage, which defines the 5′ end of a complementary piRNA with a 10-nt 5′ end overlap. Reciprocal cleavages of sense and antisense transcripts by the two partner piRNAs result in piRNA amplification (ping-pong cycle). Ping-pong cycles can be either homotypic or heterotypic (sense and antisense piRNAs are loaded into either the same protein [e.g., Mili in mice] or two different proteins [e.g., Aubergine {Aub} and Ago3 in flies]) (Aravin et al. 2007, 2008; Brennecke et al. 2007; Gunawardane et al. 2007; Zhang et al. 2011). Third, piRNAs guide PIWI clade proteins to complementary target RNAs to initiate silencing. Nuclear PIWI proteins (Miwi2 in mice and Piwi in flies) target nascent transposon transcripts and mediate local heterochromatin formation and transcriptional silencing (Shpiz et al. 2011; Wang and Elgin 2011; Sienski et al. 2012; Le Thomas et al. 2013; Rozhkov et al. 2013; Pezic et al. 2014). Cytoplasmic PIWI proteins (Mili/Miwi in mice and Aub/Ago3 in flies) elicit post-transcriptional silencing via slicer-mediated target cleavage (Gunawardane et al. 2007; Reuter et al. 2011).

In the *Drosophila* ovary, somatic cells and germline cells express piRNA pathways with different architectures. In somatic cells, all piRNAs are primary and are loaded into Piwi (Lau et al. 2009; Malone et al. 2009; Saito et al. 2009). Besides Piwi, germline cells also express Aub and Ago3. The prevailing view is that piRNA precursors are first processed into primary piRNAs that are loaded into Piwi and Aub (Brennecke et al. 2007; Senti and Brennecke 2010;

Olivieri et al. 2012). While loaded Piwi translocates to the nucleus to elicit transcriptional silencing, Aub remains cytoplasmic and cleaves target RNAs to trigger biogenesis of secondary piRNAs fueling Ago3. Piwi-bound piRNAs should therefore be independent of secondary piRNA biogenesis. Recent work, however, uncovered that secondary piRNA-guided target slicing not only fuels the ping-pong cycle but also initiates Zucchini-dependent, 3′-directed biogenesis of primary piRNAs from the remainder of the target transcript (Han et al. 2015; Mohn et al. 2015).

Here, we systematically define the interdependencies between primary and secondary piRNA biogenesis and between transcriptional and post-transcriptional TE silencing in developing *Drosophila* ovaries. We show that cytoplasmic target cleavage by Aub/Ago3 is the predominant mechanism that defines the levels and identities of the nuclear Piwi-bound piRNA pool and therefore the transcriptional silencing capacity of the pathway. We also uncover an unexpected diversity of TE silencing by the transcriptional and post-transcriptional modules of the piRNA pathway and show that tight TE repression requires the action of both silencing modules.

## Results

### An experimental system to determine the hierarchy of primary and secondary piRNA biogenesis processes in vivo

**Figure 1.**
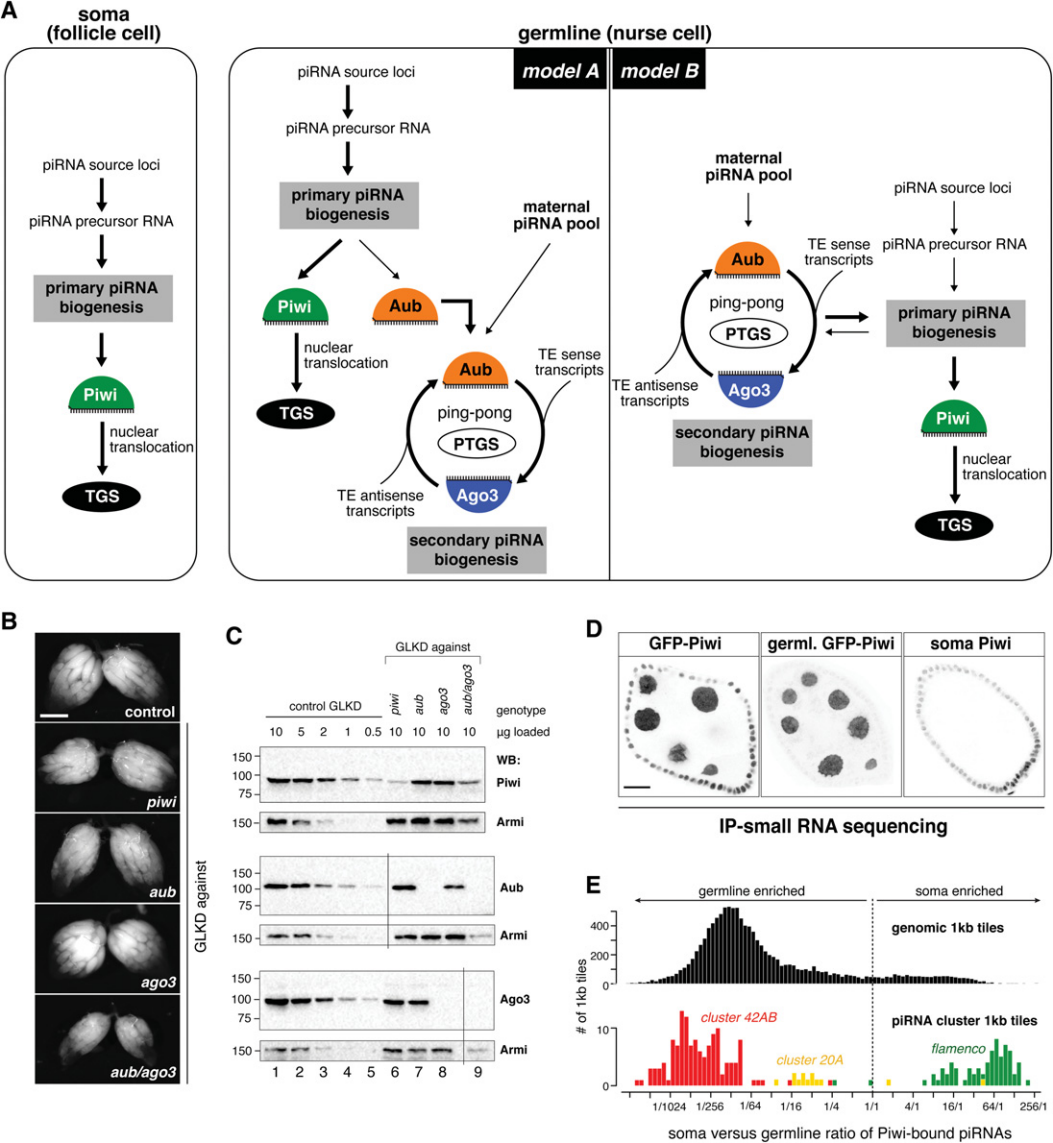
An experimental system to determine the hierarchy of primary and secondary piRNA biogenesis processes in vivo. (*A*) The cartoon shows the piRNA pathway architecture in somatic and germline cells of the *Drosophila* ovary. Two hierarchical models are depicted for the germline. (TGS) Transcriptional gene silencing; (PTGS) post-transcriptional gene silencing. (*B*) Bright-field images show the ovarian morphology of the indicated genotypes. Bar, 500 μm. (*C*) Western blot analysis of ovary lysates of control germline knockdown (GLKD; 10, 5, 2, 1, and 0.5 μg of total protein; lanes *1–5*) or the indicated GLKDs (10 μg of each; lanes *6–9*). Membranes were reprobed with anti-Armi. Vertical lines indicate spliced out marker lanes (see Supplemental Fig. S1C). (Lane *6*) Note that somatic follicle cells express Piwi in *piwi* GLKD ovaries (Supplemental Fig. S1A). (*D*) Color-inverted confocal images show GFP-Piwi fluorescence in stage 7 egg chambers that express GFP-Piwi in the soma and germline (*left*) or the germline only (*middle*). The egg chamber at the *right* is depleted for germline Piwi (GLKD) and was stained for endogenous Piwi (“soma Piwi”). (*E*) Histograms display the soma/germline ratio of Piwi-bound piRNAs (obtained from the ovaries shown in *D*) mapping uniquely to genomic 1-kb tiles (*top*) or 1-kb tiles from piRNA cluster *42AB*, *20A*, or *flamenco* (*bottom*).

In the *Drosophila* ovarian germline, primary and secondary piRNA biogenesis co-occur. Figure 1A depicts two models of how these processes could be hierarchically connected. While model A, the prevailing view in the literature, places the ping-pong cycle downstream from primary biogenesis, model B proposes a significant input from secondary piRNAs into primary biogenesis, yet the extent of this input is unclear. To experimentally test the two opposing models, we depleted Piwi, Aub, Ago3, or Aub/Ago3 together during oogenesis and determined the resulting changes in piRNA populations as well as in the transcriptional and post-transcriptional silencing capacity of the germline pathway.

In choosing a genetic perturbation system, we considered that TE expression as well as piRNA populations differ substantially between *Drosophila melanogaster* strains and between different oogenesis stages. Given this, a constant genetic background and comparable ovarian morphology are key to analyze mutant phenotypes at the molecular level. Available fly strains carrying mutant *piwi*, *aub*, or *ago3* alleles have diverse genetic backgrounds. Moreover, oogenesis is perturbed in *piwi* mutant flies, a phenotype attributable to Piwi's function in somatic support cells (Cox et al. 2000; Jin et al. 2013). We therefore depleted Piwi, Aub, or Ago3 specifically in germline cells via the shRNA-mediated knockdown system (germline knockdown [GLKD]) (Ni et al. 2011). Using this system, the resulting flies have a nearly identical genetic background, and their ovarian morphology is by and large unaffected (only Aub/Ago3 double depletion ovaries are smaller and have disintegrating late egg chambers) (Fig. 1B). Moreover, the target proteins are undetectable by immunofluorescence or Western blotting using ovarian lysate (Fig. 1C; Supplemental Fig. S1A–C). To rule out the possibility that residual low levels of the target proteins impacted our results, we generated a series of new genetic null alleles for *piwi*, *aub*, and *ago3* using CRISPR/Cas9 in an isogenic *white*^*1118*^ background. Throughout this study, we used these flies to confirm the central findings obtained with the knockdown system (see below).

To systematically characterize the germline piRNA complement in the respective genotypes, we sequenced total piRNAs and piRNA populations specifically bound to all three PIWI clade proteins. We normalized Piwi/Aub/Ago3 piRNAs to their relative cellular levels (Supplemental Fig. S1D; Mohn et al. 2015). Given that Piwi is expressed in ovarian germline and soma, we also profiled piRNAs specifically bound to germline Piwi (*nanos* promoter-driven GFP-Piwi transgene) (Fig. 1D). This allowed us to quantify germline Piwi-bound piRNA populations, which in sum account for ∼80% of all Piwi-bound piRNAs (Fig. 1E; Supplemental Fig. S1D).

### Aub and Ago3 globally define the levels and identities of germline Piwi-bound piRNAs

To examine the impact of Aub/Ago3-bound secondary piRNAs on primary piRNA biogenesis on a global scale, we compared the levels and identities of germline Piwi-bound piRNAs from ovaries depleted for Aub, Ago3, or Aub/Ago3 together with those from control ovaries.

**Figure 2.**
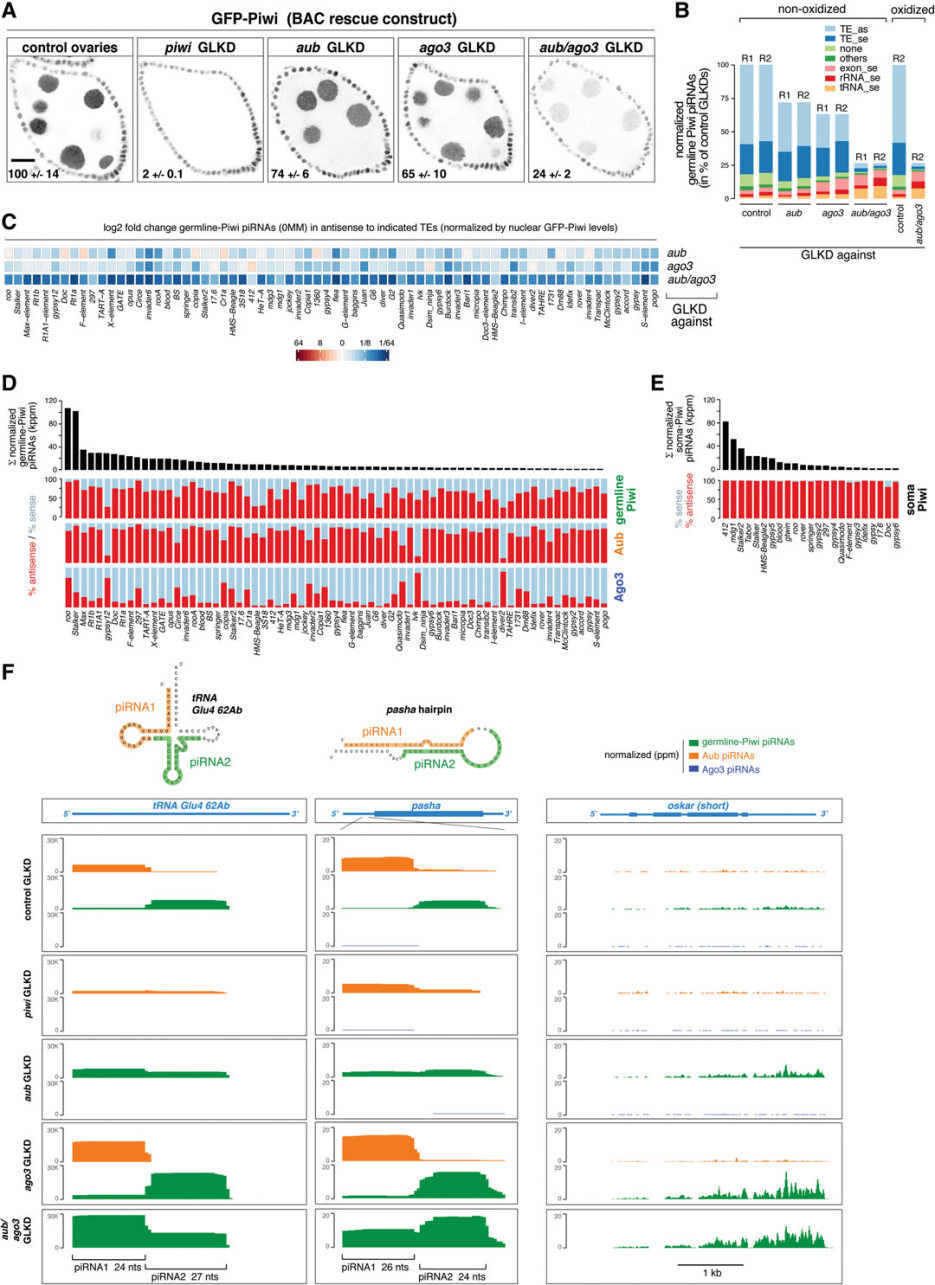
Aub and Ago3 globally define the levels and identities of germline Piwi-bound piRNAs. (*A*) Confocal images show GFP-Piwi fluorescence (BAC transgene) in stage 7 egg chambers of the indicated genotypes. Bar, 20 μm. Numbers indicate average GFP-Piwi fluorescence in nurse cell nuclei (in percentage of control value ± SD) for at least four samples per genotype. (*B*) Annotation of germline Piwi piRNA populations (normalized; in percentage of controls) derived from ovaries of the indicated genotypes. (R1/2) Biological replicates. Two R2 samples were split to generate nonoxidized and oxidized libraries. (*C*) Heat map shows the log_2_ fold changes of normalized germline GFP-Piwi piRNAs in the indicated GLKDs mapping antisense to the TEs (0MM; displayed are those TEs that make up 95% of all germline Piwi piRNAs). (*D*,*E*) Bar diagrams at the *top* show normalized levels of germline (*D*) or soma (*E*) Piwi-bound piRNAs mapping to the indicated TEs (their sum is >95% of all germline or soma Piwi-bound TE piRNAs). The plots *below* show the antisense (red) and sense (blue) bias for germline Piwi-bound, Aub-bound, Ago3-bound (*D*), and soma Piwi-bound (*E*) piRNAs. (*F*) University of California at Santa Cruz (UCSC) browser shots show normalized Aub-bound (orange), germline Piwi-bound (green), and Ago3-bound (blue) piRNAs mapping to *tRNA-Glu-CTC* (all mappers), the *pasha* hairpin, and the *oskar* mRNA (unique mappers) in the indicated GLKDs.

The failure to load Piwi with a piRNA prevents Piwi's nuclear accumulation and leads to its degradation (Olivieri et al. 2010; Saito et al. 2010). We therefore used nuclear Piwi levels as a proxy for Piwi-bound piRNA levels and thus primary piRNA biogenesis output. We expressed GFP-tagged Piwi from its endogenous regulatory control regions and quantified germline nuclear GFP-Piwi intensities in the various genotypes (GFP-Piwi intensities in follicle cells served as a normalization reference) (Fig. 2A). RNAi-mediated depletion of Piwi results in its nearly complete loss (2% ± 0.1%). Depletion of Aub or Ago3 leads to a reduction in Piwi levels to 74% ± 6% and 65% ± 10%, respectively (see also Li et al. 2009; Malone et al. 2009). Remarkably, loss of Aub and Ago3 together reduces Piwi levels to 24% ± 2%. Very similar results were obtained for endogenous Piwi levels or with a germline-specific GFP-Piwi construct (Fig. 1C; Supplemental Figs. S1A, S2A,B). The severe loss of nuclear Piwi upon Aub/Ago3 double depletion indicates that secondary piRNA-guided transcript cleavage is the dominant input into primary piRNA biogenesis fuelling Piwi.

We next characterized the population of germline Piwi-bound piRNAs that remain in ovaries depleted for Aub, Ago3, or both together (Fig. 2B). We used the respective nuclear GFP-Piwi intensities for normalization (a constant 1U bias and typical piRNA size profiles support that these are bona fide piRNAs) (Supplemental Fig. S2A–D). This demonstrates that the most sensitive piRNA populations are those derived from TE sequences. Loss of Aub/Ago3 together results in a reduction of germline Piwi-bound piRNAs mapping to TEs to 5% (antisense) and 9.5% (sense), respectively (Fig. 2B). Consistent with Piwi-bound piRNAs being dependent on transcript slicing, their levels strongly correlate with those of secondary piRNAs mapping to individual TEs in control ovaries, although total piRNA levels differ by several orders of magnitude for the various TEs (Supplemental Fig. S2E). As Aub- and Ago3-mediated cleavages both trigger primary piRNA biogenesis (Han et al. 2015; Mohn et al. 2015), these findings allude to why Piwi–bound piRNAs in the germline display only a modest TE antisense bias (average: 64%) that typically correlates with the sense/antisense bias of Aub/Ago3-bound piRNAs. In stark contrast, TE-derived piRNAs bound to Piwi in somatic follicle cells are ∼99% antisense (Fig. 2D,E). This underlines the fundamentally different piRNA biogenesis system in the soma, where piRNA biogenesis is slicer-independent, and the antisense bias is hardwired in the architecture of unistranded piRNA clusters such as *flamenco* (Malone et al. 2009).

Interestingly, absolute levels of germline Piwi-bound piRNAs derived from mRNAs (sense), tRNAs, or rRNAs are unchanged or even increased in ovaries depleted for secondary piRNAs (Fig. 2B). This points to alternative entry routes into primary piRNA biogenesis besides secondary piRNA-guided slicing. Cloning of these non-TE-derived piRNAs is oxidation-resistant, indicative of 2′ O-methylation at the 3′ end, a signature of mature bona fide piRNAs (Fig. 2B; Vagin et al. 2006). We identified several atypical piRNA sources that indicate that RNAs carrying a slicer-independent 5′ monophosphate can act as piRNA precursors in vivo. For example, some tRNAs give rise to pairs of highly defined and abundant piRNAs (Fig. 2F): While the first piRNA starts precisely at the RNase P processing site and is loaded predominantly into Aub, the second and immediately adjacent piRNA is bound mostly by Piwi. A similar pattern is seen for the microRNA (miRNA)-mimicking hairpin in the 5′ UTR of the *pasha*/*DGCR8* mRNA (Fig. 2F; Kadener et al. 2009). Depletions of Piwi or Aub indicate that these proteins compete for each other's preferred loading substrate (Fig. 2F). We propose that, in wild-type cells, Aub scavenges various RNA precursors with a 5′ monophosphate. piRNA 3′ ends are presumably generated by endonucleolytic cleavage, which simultaneously defines the 5′ end of a Piwi-bound trail-RNA—precisely as in slicer-induced piRNA biogenesis (Han et al. 2015; Mohn et al. 2015). In support of this hypothesis, *pasha*- and tRNA-derived piRNAs are Zucchini-dependent (Supplemental Fig. S2F). The population of mRNA-derived piRNAs is very diverse. In contrast to somatic cells, genic piRNAs in the germline are derived from most of the mRNA molecule and are not restricted to 3′ UTR portions (Fig. 2F, right; Robine et al. 2009). Although these piRNAs display characteristics of phased piRNAs (e.g., downstream U bias) (Han et al. 2015; Mohn et al. 2015), we could not detect any pattern indicative of how these mRNAs are funneled into biogenesis.

Taken together, our results demonstrate that Aub/Ago3-mediated cleavage is the major trigger for primary piRNA biogenesis fueling germline Piwi but that alternative entry routes that feed into primary piRNA biogenesis exist.

### Primary processing of piRNA cluster transcripts depends on slicing

**Figure 3.**
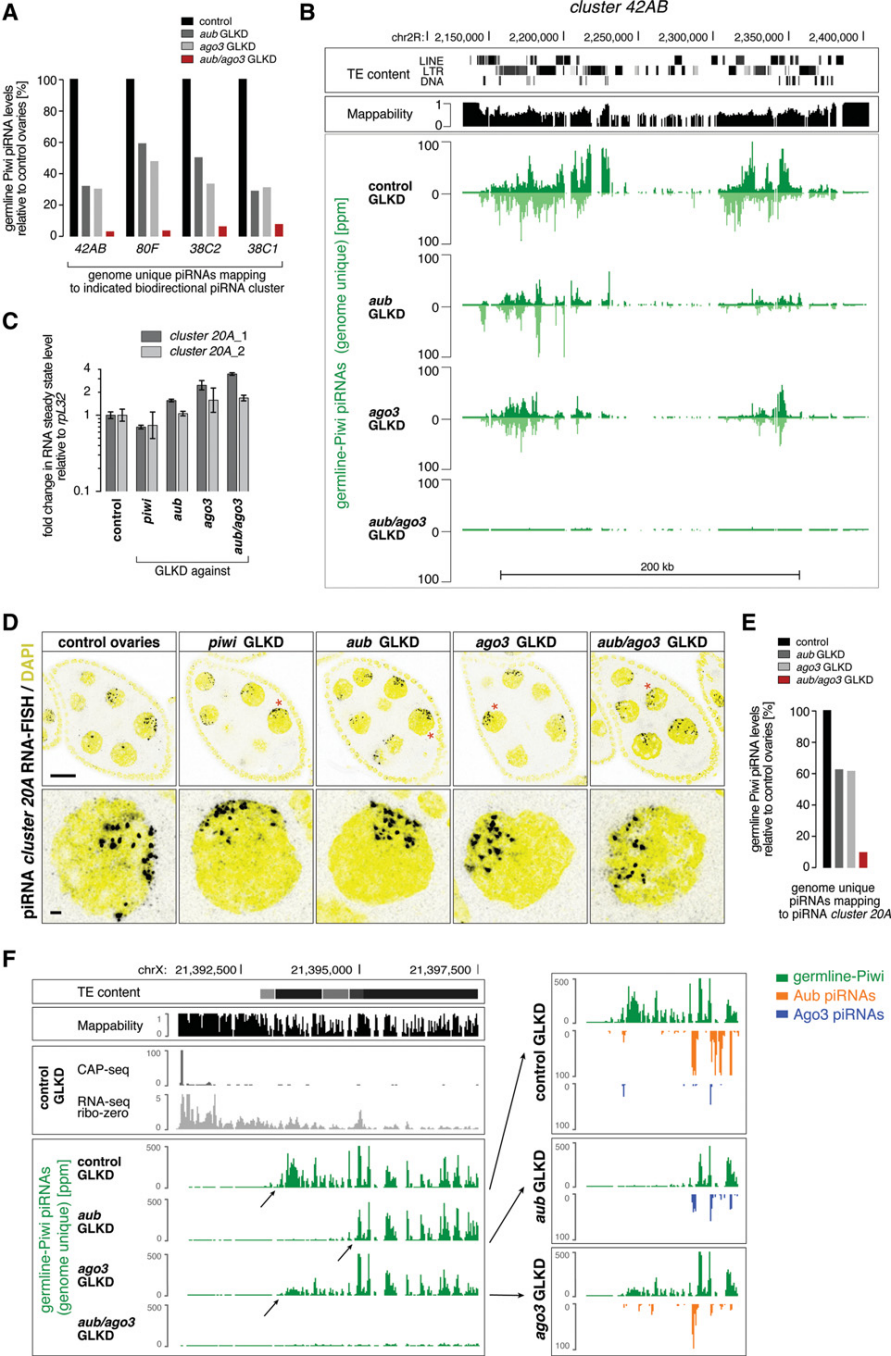
Aub/Ago3-mediated cleavage specifies piRNA cluster transcripts as substrates for primary piRNA biogenesis. (*A*) Shown are normalized levels of germline Piwi-bound piRNAs (in percentage of controls) obtained from the ovaries of the indicated genotypes mapping uniquely to major dual-strand piRNA clusters. (*B*) The UCSC browser shot shows normalized germline Piwi-bound piRNA profiles mapping to cluster *42AB*. The *top* tracks display TE content and a mappability index of 25mers. (*C*) Shown are the fold changes of cluster *20A* transcript levels (relative to *rpL32*) in the indicated GLKDs by two independent qPCR amplicons. *n* = 3. Error bars indicate SD. (*D*) Color-inverted confocal images of stage 7 egg chambers (bars, 20 μm) show cluster *20A* RNA-FISH (black) and DAPI (yellow). Red asterisks mark single nuclei shown enlarged *below*. Bars, 2 μm. (*E*) Bar diagram indicating the normalized levels of germline Piwi-bound piRNAs (obtained from ovaries of the indicated genotypes) mapping uniquely to cluster *20A*. (*F*) A UCSC browser screenshot as in *B* depicts the 5′ end of piRNA cluster *20A*. The Cap-seq and ribozero RNA sequencing (RNA-seq) tracks indicate the position of the transcriptional start site of this unistranded cluster. The green graphs show normalized germline Piwi-bound piRNA populations (genome-unique mappers) obtained from ovaries of the indicated genotypes (arrows indicate initiation sites of piRNA biogenesis). In addition, the *right* panels show Aub-bound (orange) and Ago3-bound (blue) piRNA populations (all mappers; only antisense) that map to this locus.

Transcripts from piRNA clusters are postulated to carry signals that specify them for primary piRNA biogenesis. We challenged this model by asking whether processing of piRNA cluster transcripts instead depends on piRNA-guided cleavage. We determined levels of germline Piwi-bound piRNAs mapping to piRNA cluster loci in control ovaries and ovaries depleted for Aub, Ago3, or Aub/Ago3 together. Remarkably, loss of Aub or Ago3 individually reduces Piwi-bound piRNAs from clusters twofold to fourfold, and loss of both factors together reduces them 10-fold to 30-fold (Fig. 3A,B). A potential caveat in this analysis is that the definition and transcription of most germline piRNA clusters partially depend on Piwi (via specifying chromatin occupancy of the Rhino–Deadlock–Cutoff complex) (Mohn et al. 2014). We therefore focused on the unistrand cluster *20A,* which is transcribed independently of Piwi and Rhino (Klattenhoff et al. 2009; Mohn et al. 2014). Indeed, cluster *20A* transcript levels and nascent transcription foci (visualized by single-molecule RNA fluorescent in situ hybridization [FISH]) are unchanged or even increased in ovaries depleted for Aub, Ago3, or both together (Fig. 3C,D). Nevertheless, levels of germline Piwi-bound cluster *20A* piRNAs drop below 10% in ovaries depleted of secondary piRNAs (Fig. 3E). Interestingly, we observed distinct piRNA biogenesis initiation sites at the 5′ end of cluster *20A* in ovaries lacking Aub or Ago3 individually (Fig. 3F, left). Inspecting the remaining Aub- or Ago3-bound piRNAs antisense to the cluster *20A* transcript (by definition *trans*-acting piRNAs originating from other sites in the genome) reveals a striking correlation between the remaining secondary piRNAs and primary biogenesis initiation sites (Fig. 3F, right).

Taken together, processing of piRNA cluster transcripts into primary piRNAs depends almost exclusively on secondary piRNA-guided cleavages. This indicates that slicing, and not transcript origin, is the specificity signal that selects transcripts for piRNA biogenesis in the ovarian germline.

### Aub/Ago3-mediated transcript cleavage defines Piwi's transcriptional silencing repertoire

We next asked whether changes in Piwi-bound piRNA levels caused by loss of secondary piRNA populations translate into TE derepression at the transcriptional level. We performed RNA polymerase II (Pol II) chromatin immunoprecipitation (ChIP) combined with deep sequencing (ChIP-seq) from control ovaries and ovaries with germline-specific piRNA pathway perturbations. We restricted the analysis to TE promoter regions, which—if active—display a pronounced Pol II peak that coincides with the transcription start site (TSS), as evidenced by a 5′ Cap-seq analysis (Supplemental Fig. S3A).

**Figure 4.**
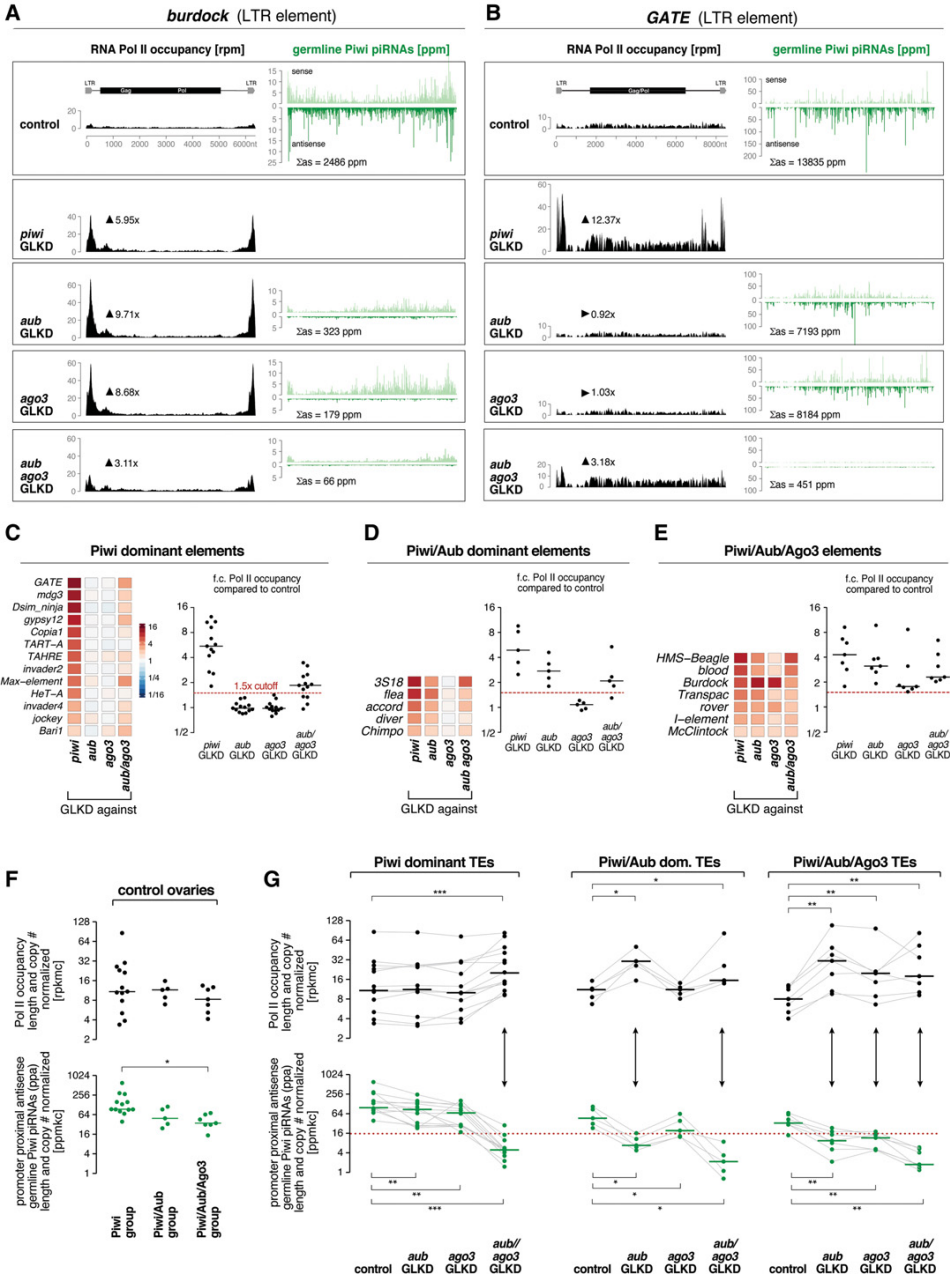
Loss of Aub or Ago3 results in transcriptional derepression of subsets of TEs by impacting levels of Piwi-bound piRNAs. (*A*,*B*) Shown are profiles of RNA Pol II occupancy and normalized germline Piwi-bound piRNAs mapping to *burdock* (*A*) or *GATE* (*B*) from ovaries of the indicated genotypes. Arrowheads indicate fold changes in Pol II occupancy at respective TE promoters, and “Σas” reports the sum of antisense piRNAs. Reduced de-repression of *burdock* in Aub/Ago3 double depletions versus Aub and Ago3 single depletions is probably due to compromised ovarian development in *aub/ago3* GLKD ovaries. (*C*–*E*) Heat maps (*left*) and jitter plots with median bars (*right*) show fold changes of Pol II occupancy at TE promoters in the ovaries of the indicated genotypes. TEs were grouped into Piwi-dominant (*C*), Piwi/Aub-dominant (*D*), and Piwi/Aub/Ago3 (*E*). (*F*,*G*) Dot plots with median bars indicating Pol II occupancy at TE promoters (black; in reads per kilobase per million and TE copy number [RPKMC]) and promoter-proximal antisense (PPA) piRNA levels (green; in parts per million per kilobase and copy number [PPMKC]) in the indicated genotypes. TEs were grouped as in *C*–*E*. Lines connect data points of individual TEs in different genotypes. Asterisks indicate significance by unpaired, one-tailed *t*-test with *P*-value ([*] *P* < 0.05) (*F*) and one-tailed Wilcoxon matched pairs signed rank test ([*] P < 0.05; [**] P < 0.01; [***] P < 0.001) (*G*). The dotted red line marks a hypothesized piRNA threshold required for efficient transcriptional silencing.

Remarkably, loss of Aub or Ago3 leads to elevated transcription of several TEs to levels comparable with those in Piwi-depleted ovaries. For example, the LTR element *Burdock* exhibits strong transcriptional increases upon loss of Aub or Ago3 (Fig. 4A). In contrast, the LTR element *GATE*, the most derepressed TE in Piwi-depleted ovaries, does not exhibit increased Pol II occupancy in ovaries lacking Aub or Ago3 (Fig. 4B). In both cases, changes in Pol II occupancy correlate with changes in antisense piRNAs bound to germline Piwi. While these are reduced ∼10-fold in the case of *Burdock*, only mild losses (1.7-fold to 1.9-fold) (Fig. 4A,B) are observed in the case of *GATE*. Here, only the simultaneous loss of Aub and Ago3 has a severe impact on Piwi-bound piRNAs (>30-fold loss), which coincides with increased *GATE* transcription (Fig. 4B).

These observations indicate that the cytoplasmic Aub and Ago3 proteins define Piwi-bound piRNA levels and hence transcriptional silencing but in a TE-specific manner. To systematically study this, we focused on the 27 active TE families in which our data confidently indicate that there is piRNA pathway-dependent transcriptional silencing in the germline (Supplemental Fig. S3B,D). All but one of these are transcriptionally derepressed in Piwi-depleted ovaries, confirming the central role of Piwi in orchestrating transcriptional silencing (Fig. 4C–E). Almost half of the Piwi-repressed elements (12 out of 27) are transcriptionally derepressed in Aub-depleted ovaries, and, of those 12, seven also require Ago3 for transcriptional silencing. Based on a cutoff of >1.5-fold increased Pol II occupancy, we divided TEs into a Piwi-dominant group (*n* = 13), a Piwi/Aub-dominant group (*n* = 5), and a Piwi/Aub/Ago3 group (*n* = 7) (Fig. 4C–E).

To test whether transcriptional derepression generally correlates with reduced levels of Piwi-bound piRNAs, we considered the recent finding that the absolute number of Piwi-bound piRNAs antisense to the nascent target transcript—in particular to TSS-proximal regions—defines Piwi's transcriptional silencing capacity (Post et al. 2014). We determined the approximate copy number of each TE in the genetic background used for this study. For each TE family, we then calculated the length and copy number-corrected level of Pol II occupancy (in reads per kilobase per million reads and per TE copy number [RPKMC], approximating transcriptional output) and promoter-proximal antisense piRNA levels normalized to 1 million sequenced miRNAs (promoter-proximal antisense [PPA] piRNAs in parts per million per kilobase and per copy number [PPMKC]).

In control ovaries, Pol II occupancy for TEs belonging to the three groups was similar overall (Fig. 4F, top), but levels of Piwi-bound antisense piRNAs differed (Fig. 4F, bottom): Piwi/Aub and especially Piwi/Aub/Ago3 elements are targeted by a considerably lower amount of piRNAs (Ø = 62 PPMKC and Ø = 42 PPMKC, respectively) compared with the Piwi-dominant group (Ø = 167 PPMKC). Given that a minimum amount of Piwi-bound piRNAs is required for transcriptional silencing (Post et al. 2014), the difference in wild-type piRNA levels (Fig. 4F) correlates with the different sensitivities of TEs to pathway perturbations (Fig. 4G). For TEs in the Piwi/Aub/Ago3 and Piwi/Aub-dominant groups, which have comparably low numbers of piRNAs per TE, depletion of Aub or Ago3 is sufficient for derepression (Fig. 4G). In contrast, piRNA levels for TEs belonging to the Piwi-dominant group—although reduced in ovaries depleted for Aub or Ago3 individually—are still higher than the piRNA levels targeting the other two groups in control ovaries. Only the simultaneous loss of Aub and Ago3 leads to severe reductions of Piwi-bound piRNAs and a concomitant transcriptional derepression of TEs similar to the other groups (Fig. 4G).

Taken together, cytoplasmic Aub/Ago3-mediated post-transcriptional silencing feeds back onto transcriptional silencing of target TEs via defining the pool of nuclear Piwi-bound piRNAs. However, different TEs display different sensitivities toward loss of Aub or Ago3. We hypothesized that this is caused by the variable dependencies of the respective secondary piRNA populations on the presence of Aub and/or Ago3.

### Obligatory heterotypic Aub–Ago3 ping-pong controls Piwi/Aub/Ago3 elements

**Figure 5.**
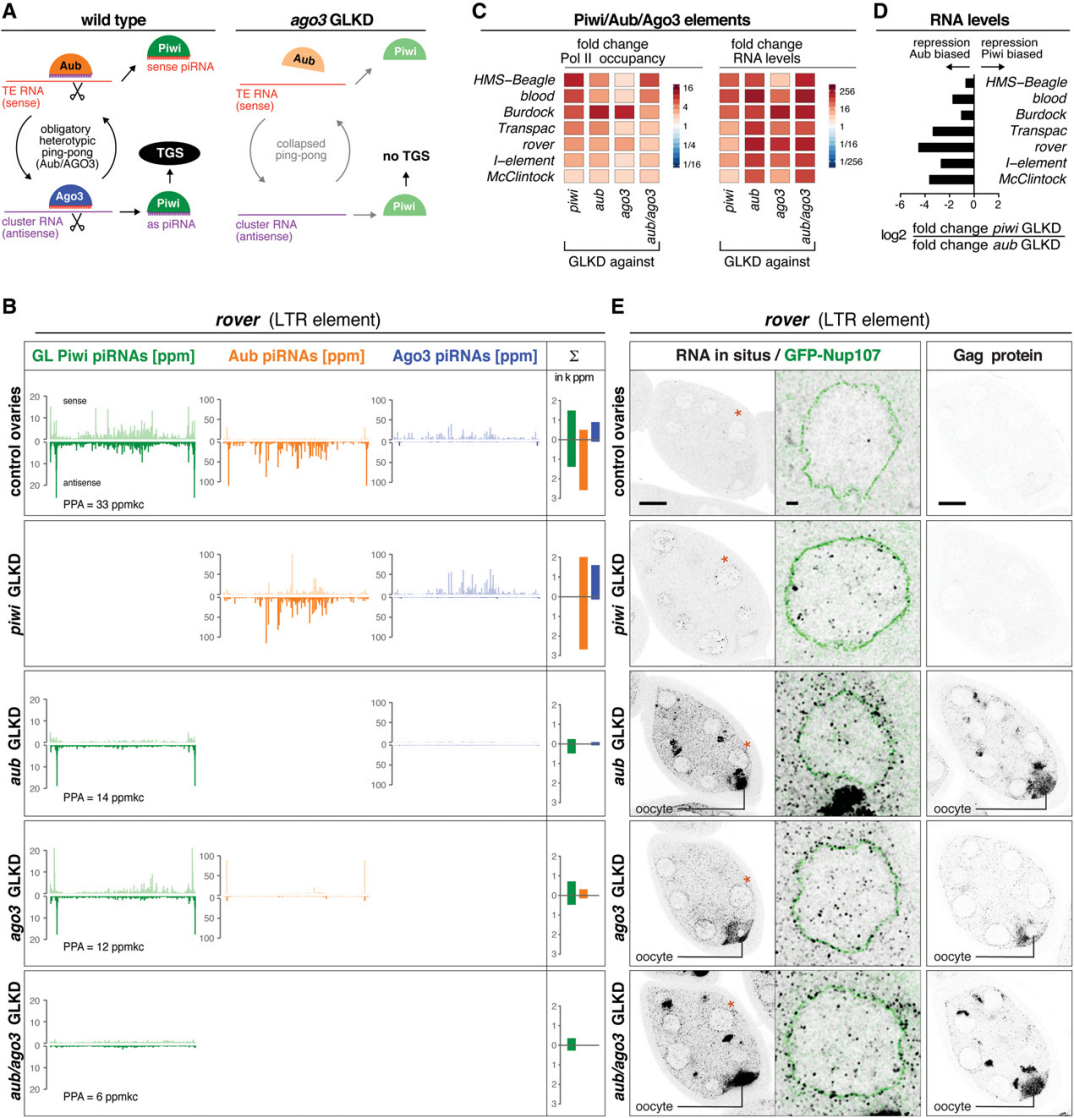
Obligatory heterotypic ping-pong defines Piwi-bound piRNAs for Piwi/Aub/Ago3 TEs. (*A*) Cartoon of piRNA biogenesis architecture for Piwi/Aub/Ago3 TEs in wild-type or Ago3-depleted ovaries. (*B*) Normalized profiles of piRNAs bound to germline Piwi (green), Aub (orange), or Ago3 (blue) mapping to *rover* are shown for the indicated genotypes (the sum of PPA germline Piwi-bound piRNAs is indicated). The bar diagrams display the respective sum of piRNA populations (sense and antisense, in 1000 × parts per million [ppm]). (*C*) Heat maps showing fold changes in Pol II occupancy at promoters or in steady-state RNA levels (sense) for the indicated TEs in the ovaries of the indicated genotypes. (*D*) Bar diagram displaying the log_2_ ratios of fold changes in steady-state RNA levels (sense) in *piwi* GLKD versus *aub* GLKD ovaries. (*E*) Color-inverted confocal images of stage 7 egg chambers depict *rover* RNA-FISH and *rover*-Gag immunolocalization in the ovaries of the indicated genotypes. Bar, 20 μm. Red asterisks mark single nuclei shown in the enlarged images (bar, 2 μm) with RNA-FISH (black) and GFP-Nup107-outlined nuclei (green).

TEs of the Piwi/Aub/Ago3 group are expected to engage in an obligatory heterotypic ping-pong cycle that requires both Aub and Ago3 (Fig. 5A). Loss of either protein would result in a collapse of secondary piRNAs and the inability to trigger primary piRNA biogenesis. Indeed, Aub/Ago3-bound piRNA populations mapping to these TEs depend on both Aub and Ago3, and the remaining piRNAs show greatly reduced ping-pong signatures (Fig. 5B; Supplemental Fig. S4A,B,E). Consequently, primary piRNA biogenesis that fuels Piwi is impaired, resulting in transcriptional derepression of these TEs (Fig. 5C). Loss of Aub or Ago3 therefore leads to the simultaneous loss of transcriptional and post-transcriptional silencing. On the other hand, loss of Piwi typically does not impair secondary piRNA biogenesis, which leaves post-transcriptional silencing unaffected or only partially impaired (Fig. 5B; Supplemental Fig. S4A,B). This predicts that TE desilencing at the RNA level is more severe in Aub- or Ago3-depleted ovaries compared with Piwi-depleted ovaries. Indeed, steady-state RNA levels of all seven TEs are twofold to 20-fold higher in Aub-depleted versus Piwi-depleted ovaries (Fig. 5D).

To examine this in more detail, we performed single-molecule RNA-FISH for *rover*, *burdock*, and the *I* element in developing ovaries. For *rover* and the *I* element, we also visualized TE-encoded proteins by immunofluorescence (Fig. 5E; Supplemental Fig. S4C,D). Transcriptional derepression of *rover* in ovaries depleted for Piwi, Aub, or Ago3 coincides with an increased number of nuclear *rover* RNA-FISH foci (presumably nascent sites of transcription). In contrast, cytoplasmic *rover* RNA and *rover* Gag protein only accumulate in nurse cells and the oocytes of ovaries depleted for Aub or Ago3 but not for Piwi (Fig. 5E). In Piwi-depleted ovaries, *rover* transcription is derepressed, but the intact secondary piRNA biogenesis machinery efficiently cleaves *rover* transcripts upon nuclear export, and triggered primary sense piRNAs are loaded into Aub (Fig. 5B,E). Similar results were obtained for the *I* element (Supplemental Fig. S4C). In the case of *burdock*, loss of Piwi partially impairs the ping-pong cycle (Supplemental Fig. S4B). Consequently, *burdock* RNA accumulates in the cytoplasm and the developing oocyte but to a lower extent than in ovaries depleted for Aub or Ago3 (Supplemental Fig. S4D).

Taken together, TEs belonging to the Piwi/Aub/Ago3 group display a strict heterotypic ping-pong cycle that consumes TE transcripts in the cytoplasm and defines the Piwi-bound piRNA pool for additional transcriptional silencing.

### Homotypic Aub/Aub ping-pong triggers primary piRNA biogenesis in the absence of Ago3 for Piwi/Aub-dominant transposons

**Figure 6.**
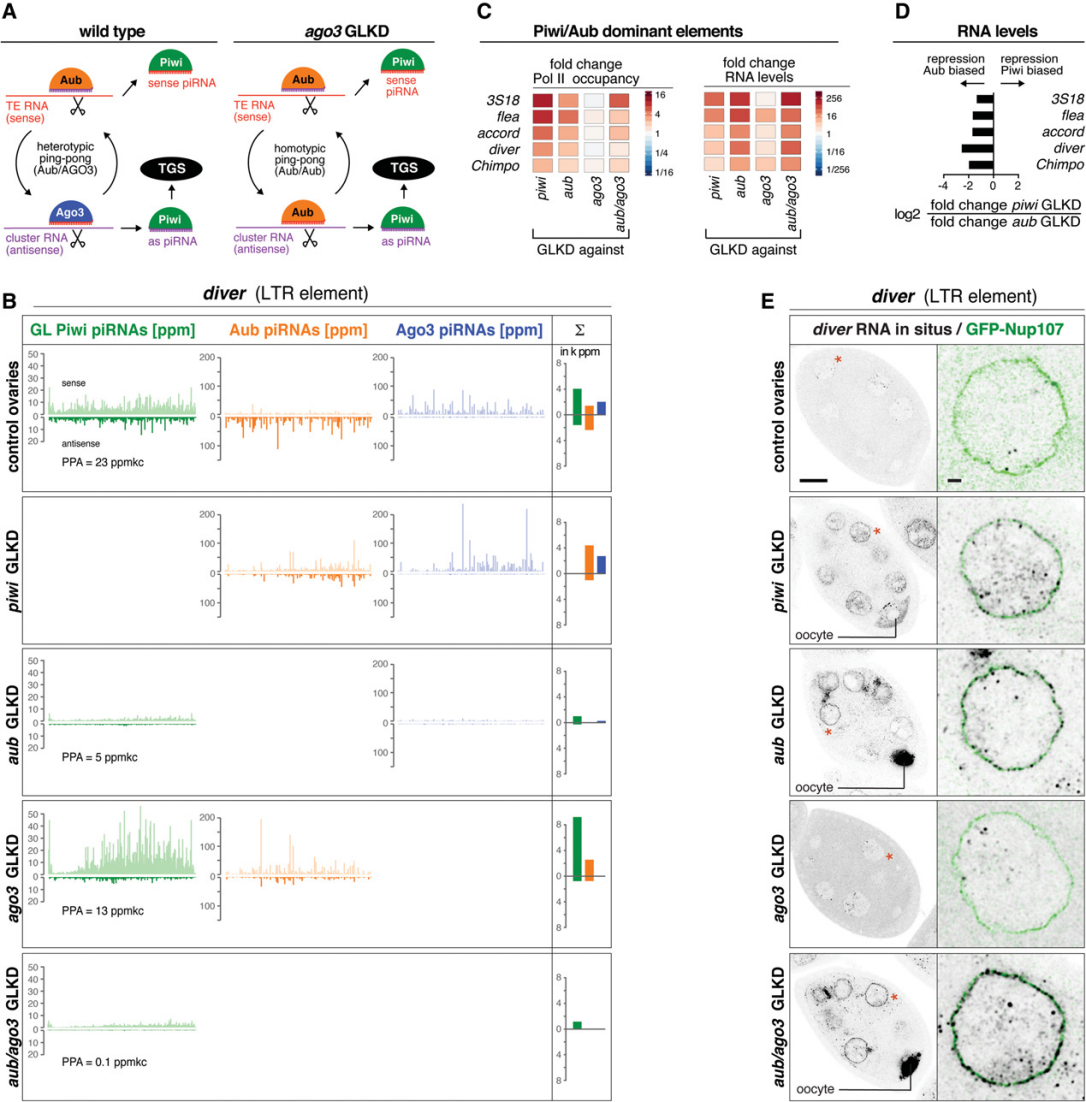
Homotypic Aub/Aub ping-pong bypasses Ago3 loss for the Piwi/Aub-dominant TEs. (*A*) Cartoon of piRNA biogenesis architecture for Piwi/Aub-dominant TEs in wild-type or Ago3-depleted ovaries. (*B*) Normalized profiles of piRNAs bound to germline Piwi (green), Aub (orange), or Ago3 (blue) mapping to *diver* are shown for the indicated genotypes (the sum of PPA germline Piwi-bound piRNAs is indicated). Bar diagrams display the respective sums of piRNA populations (sense and antisense; in 1000× ppm). (*C*) Heat maps show fold changes in Pol II occupancy at promoters or in steady-state RNA levels (sense) for the indicated TEs in the ovaries of the indicated genotypes. (*D*) The bar diagram displays the log_2_ ratios of fold changes in steady-state RNA levels (sense) in *piwi* GLKD versus *aub* GLKD ovaries. (*E*) Color-inverted confocal images of stage 7 egg chambers depict *diver* RNA-FISH in the ovaries of the indicated genotypes. Bar, 20 μm. Red asterisks mark single nuclei shown in the enlarged images (bar, 2 μm) with RNA-FISH (black) and GFP-Nup107-outlined nuclei (green).

The existence of a Piwi/Aub-dominant group of TEs whose transcriptional repression requires Aub but not Ago3 predicts that secondary piRNA biogenesis for these elements switches from heterotypic ping-pong to the homotypic Aub/Aub cycle in the absence of Ago3 (Fig. 6A; Li et al. 2009). Indeed, loss of Ago3 affects ping-pong of the Piwi/Aub elements on average much less than that of Piwi/Aub/Ago3 elements. This results in robust Aub sense/antisense piRNA profiles with the characteristic 10-nt offset (Fig. 6B; Supplemental Fig. S5A,B). In contrast, loss of Aub leads to a collapse of Ago3-bound piRNA populations and a loss of ping-pong for Piwi/Aub-dominant elements—indistinguishable from the Piwi/Aub/Ago3 elements (Fig. 6B; Supplemental Fig. S5A,B). Consistently, Piwi-bound piRNA populations are more dependent on Aub than on Ago3 (Figs. 4G, 6B). TEs of the Piwi/Aub-dominant group are therefore wired more robustly into the ping-pong cycle in that Aub/Aub homotypic ping-pong compensates for Ago3 loss and provides sufficient slicer cleavage events to trigger primary piRNA biogenesis.

TEs in the Piwi/Aub-dominant group also display higher levels of steady-state RNA in Aub-depleted versus Piwi-depleted ovaries (Fig. 6D,E; Supplemental Fig. S5C). Consistent with this, the ping-pong cycle is only partially impaired upon Piwi loss (Fig. 6B; Supplemental Fig. S5B), allowing for variable degrees of post-transcriptional silencing. As a consequence, TE RNAs accumulate significantly more in nurse cell and oocyte cytoplasm in Aub-depleted than in Piwi-depleted ovaries (Fig. 6E; Supplemental Fig. S5C). Loss of Ago3 instead does not lead to increased transcription and only weakly increases steady-state RNA levels.

### Simultaneous loss of Aub/Ago3 impairs primary piRNA biogenesis for transposons of the Piwi-dominant group

**Figure 7.**
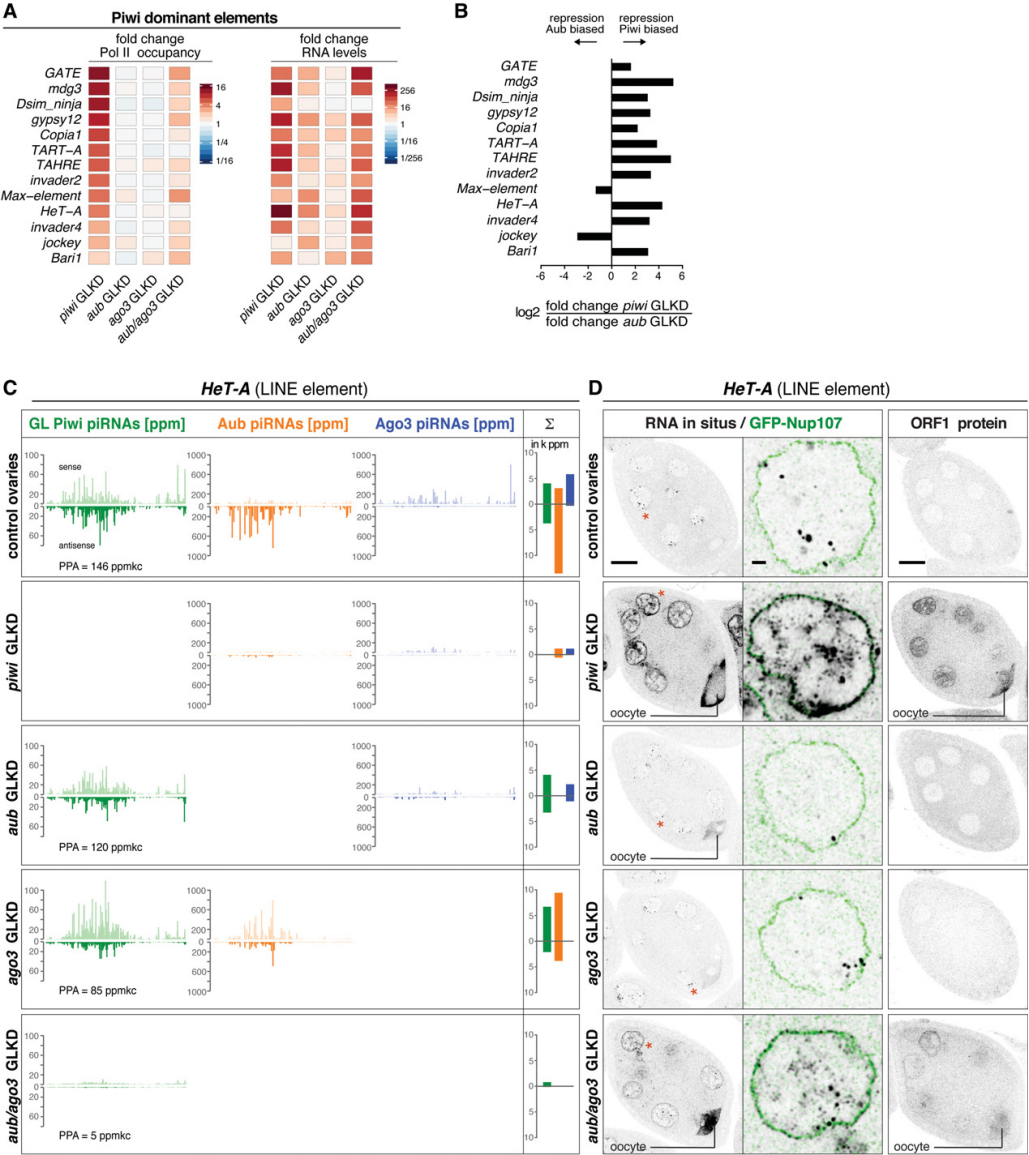
The simultaneous loss of Aub and Ago3 blocks primary piRNA biogenesis for Piwi-dominant elements. (*A*) Heat maps showing fold changes in Pol II occupancy at promoters or in steady-state RNA levels (sense) for the indicated TEs in ovaries of the indicated genotypes. (*B*) Bar diagrams display the log_2_ ratios of fold changes in steady-state RNA levels (sense) in *piwi* GLKD versus *aub* GLKD ovaries. (*C*) Normalized profiles of piRNAs bound to germline Piwi (green), Aub (orange), or Ago3 (blue) mapping to *HeT-A* are shown for the indicated genotypes (the sum of PPA germline Piwi-bound piRNAs is indicated). Bar diagrams display the respective sums of piRNA populations (sense and antisense; in 1000× ppm). (*D*) Color-inverted confocal images of stage 7 egg chambers depict *HeT-A* RNA-FISH or *HeT-A* ORF1 immunolocalization in the ovaries of the indicated genotypes. Bar, 20 μm. Red asterisks mark single nuclei shown in enlarged images (bar, 2 μm) with RNA-FISH (black) and GFP-Nup107-outlined nuclei (green).

Transcription of the Piwi-dominant group of TEs increases above threshold only upon loss of Piwi but not upon loss of Aub or Ago3. In addition, at the steady-state RNA level, all but two of these elements show strongest derepression upon loss of Piwi (Fig. 7A,B). These data would be compatible with primary piRNA biogenesis being independent of secondary piRNAs for these TEs. However, Piwi-dominant elements display patterns of homotypic Aub/Aub ping-pong in Ago3-depleted ovaries, and loss of Aub results in considerably milder losses of Ago3-bound piRNA levels compared with elements of the other two groups (Fig. 7C; Supplemental Fig. S6A,B,E). Remarkably, depletion of Aub and Ago3 together leads to transcriptional derepression of Piwi-dominant TEs and elevated TE steady-state RNA levels that approximate those seen in Piwi-depleted ovaries (Fig. 7A). This is accompanied by a strong reduction in germline Piwi-bound piRNAs mapping antisense to TEs (average fold loss: 22 ± 11) (Fig. 4G), strongly supporting the notion that, even in this group, Piwi-bound piRNAs are defined by cytoplasmic Aub/Ago3-mediated cleavage.

For a detailed analysis, we selected *HeT-A*, *Max*, and *mdg3*. These TEs represent contrasting examples of how depletion of Piwi affects secondary piRNA biogenesis: While Piwi loss nearly eliminates secondary piRNAs for *HeT-A* and *mdg3,* it leaves ping-pong unaffected for *Max* (Fig. 7C; Supplemental Fig. S6A,B). Consistent with the Pol II ChIP data, increases in nuclear RNA-FISH signal are only detectable upon loss of Piwi or Aub/Ago3 together but not when Aub or Ago3 is depleted individually (Fig. 7D; Supplemental Fig. S6C,D). On the other hand, cytoplasmic accumulation of TE transcripts as well as TE-encoded proteins depend on the impact of Piwi loss on secondary piRNA populations: In the case of *HeT-A* and *mdg3*, RNA and protein levels strongly increase in the cytoplasm of Piwi-depleted ovaries (Fig. 7D; Supplemental Fig. S6C). In contrast, cytoplasmic *Max* transcripts do not accumulate upon Piwi loss. Only in Aub/Ago3 double depletions, when both transcriptional and post-transcriptional silencing are defective, do *Max* transcripts accumulate in the cytoplasm and oocyte (Supplemental Fig. S6D).

Taken together, TEs of the Piwi-dominant group are very robustly wired into secondary piRNA biogenesis. Only the simultaneous loss of Aub and Ago3 reveals that, also for this group, secondary piRNA populations are the major specificity signal for the generation of Piwi-bound piRNAs in the germline.

We speculated that the robustness of Piwi-dominant TEs is based on their much longer evolutionary history within the *D. melanogaster* genome. To test this, we determined the approximate sequence divergence of all TEs in the genome. We determined the amount of piRNAs mapping with zero versus three mismatches to the TE consensus sequence (which resembles the active element) in control ovaries. This shows that piRNAs matching TEs from the Piwi/Aub-dominant and the Piwi/Aub/Ago3 groups match consensus sequences closely, reflecting evolutionarily young TEs. In contrast, piRNAs derived from many Piwi-dominant TEs are much more diverged, suggesting that these are evolutionarily older TEs (Supplemental Fig. S7A–F). We speculate that older TEs have more insertions in piRNA-producing loci, making them more robust toward genetic perturbations in the ping-pong cycle.

### Independent genetic support for the validity of the GLKD system

Our study is based on germline-specific, transgenic RNAi (Ni et al. 2011). While very efficient, it is possible that residual target protein levels affect data interpretation. We therefore generated new alleles of Piwi, Aub, and Ago3 in an isogenic *white*^*1118*^ background using CRISPR/Cas9 (Jinek et al. 2012; Gokcezade et al. 2014). The isolated frameshift mutations result in early translational stops. Immunofluorescence and Western blot analysis underscore that the novel allelic combinations are molecular nulls (Supplemental Fig. S8A–D). The phenotypic analyses of the novel alleles coincide with the results obtained with the knockdown system: Loss of Piwi does not affect Aub/Ago3 localization to nuage. Loss of Aub prevents Ago3 localization to nuage, and loss of Ago3 does not affect Aub localization to nuage (Supplemental Fig. S8D). Importantly, ovaries lacking Aub, Ago3, or Aub/Ago3 together exhibit reduced Piwi levels in the germline to an extent that is very similar to the results obtained with the knockdown system (Supplemental Fig. S8 D–F). Finally, patterns of TE derepression mirror those observed using germline-specific RNAi (Supplemental Fig. S8G). Hence, in all aspects, the genetic null alleles support the results obtained with the knockdown system.

## Discussion

Highly diverse piRNA populations are processed from a limited set of single-stranded precursor transcripts. How the cell ensures that these but not other, often much more abundant, RNAs enter piRNA biogenesis is a central yet unresolved question. At some level, precursor selection, which defines the target spectrum of the pathway, must discriminate self (endogenous mRNAs) versus nonself (TE RNA) transcripts. Here we show that transcript slicing guided by secondary piRNAs is the major specificity signal that defines the population of secondary as well as primary piRNAs in the *Drosophila* ovarian germline. As Aub/Ago3-mediated cleavage requires extensive complementarity between piRNAs and target RNAs (Wang et al. 2014; Mohn et al. 2015), this setup exploits one of the central differences between TE transcripts and endogenous mRNAs; namely, at some point following a TE invasion, cells express TE sense and antisense transcripts. Therefore, the key reason why piRNA cluster transcripts but not genic mRNAs are efficient piRNA biogenesis substrates does not lie in their transcript history but rather in the different likelihoods of being cleaved by Aub/Ago3. Of note, a few mRNAs do harbor TE sequence stretches and therefore piRNA target sites (typically in their 3′ UTRs). As expected, the corresponding mRNA portions as well as the downstream sequences are significant piRNA sources (Shpiz et al. 2014; Mohn et al. 2015). In the following, we discuss central aspects that are connected to our work.

### Interdependencies between transcriptional and post-transcriptional TE silencing

The adaptive nature of secondary piRNA biogenesis (ping-pong cycle) is considered to be a central feature of the piRNA pathway (Brennecke et al. 2007). Our findings extend this concept to the nuclear transcriptional silencing module: Cytoplasmic slicing of TE messages and cluster transcripts defines the pool of Piwi-bound piRNAs that are required for efficient transcriptional silencing. This represents a classic feedback system in which an increase in TE expression would result in increased cytoplasmic slicing, which in turn results in increased Piwi-bound piRNAs and hence tighter transcriptional target repression.

As Piwi also specifies TE insertions as piRNA source loci via the Rhino–Deadlock–Cutoff complex (Mohn et al. 2014), sense and antisense transcripts serving as piRNA biogenesis substrates are still produced despite silencing of canonical TE transcription. This may also explain why loss of Piwi has a strong impact on secondary piRNA biogenesis for a subset of TEs. We hypothesize that, in these cases (e.g., *mdg3* and *burdock*), TE sequences have not yet been hardwired into major piRNA clusters (which are maintained Piwi-independently) but that stand-alone insertions (where Piwi is essential to recruit Rhino) serve as the major sources of antisense transcripts to continuously fuel ping-pong.

The syncytial architecture of the female *Drosophila* germline—where nurse cells supply all RNA and protein to the growing oocyte through cytoplasmic bridges—highlight the biological significance of the post-transcriptional silencing branch. This is particularly evident in Piwi-depleted ovaries and for those TEs (e.g., *rover* or *Max*) where loss of Piwi does not impair the ping-pong cycle. Transcripts and proteins from these TEs—despite maximal transcriptional derepression—do not or only very mildly accumulate in the oocyte, preventing efficient TE transposition. In contrast to this, we observed maximal TE RNA and protein accumulation in ovaries lacking Aub and Ago3 together, where transcriptional and post-transcriptional silencing are both impaired.

What underlies the different dependencies of the various TEs on Aub and Ago3 in terms of maintaining a ping-pong cycle is mostly unclear. Our analysis of TE sequence divergence suggests that TEs of the Piwi/Aub-dominant and Piwi/Aub/Ago3 groups have colonized the *D. melanogaster* genome more recently and are more sensitive to pathway perturbations. In contrast, many Piwi-dominant TEs are present as older insertions in the genome and might have populated many piRNA source loci, thereby exhibiting robust ping-pong patterns. Alternatively, the different absolute levels of sense/antisense precursor transcripts impinge on ping-pong robustness.

### The initiation of piRNA biogenesis and the role of maternally inherited piRNAs

Our data attest a central role to transcript slicing in specifying the diverse primary and secondary piRNA populations in the ovarian germline (Han et al. 2015; Mohn et al. 2015). Much less clear is how this process is initiated during development. In this respect, maternally deposited piRNAs (especially those bound to Aub) (Brennecke et al. 2008) that are enriched in developing primordial germ cells of the embryo are likely to play a central role in kick-starting the ping-pong cycle but also in defining the target spectrum of nuclear Piwi.

How primary piRNA biogenesis in *Drosophila* ovarian somatic cells is steered to piRNA cluster transcripts and a subset of cellular mRNAs is a central, unresolved question. Our data indicate that alternative, slicer-independent ways to initiate piRNA biogenesis (e.g., from tRNA precursors) (Fig. 2F) do exist—even in wild-type germline cells. It is currently unclear to what extent the slicing-mediated trigger process shapes piRNA populations during mammalian spermatogenesis. Remarkably, however, the murine nuclear PIWI clade protein Miwi2 directly receives responder piRNAs after Mili-mediated transcript cleavage (Aravin et al. 2008). This uncovers an intriguing conceptual similarity between the fly and mouse pathways in that the piRNA pool loaded into the respective nuclear PIWI clade protein is defined by prior piRNA-guided transcript cleavage and therefore responds to the expression of active TEs.

All in all, our data provide a systematic understanding of piRNA populations in the *Drosophila* ovarian germline and synthesize several previously unconnected findings into a coherent model of TE repression. In the broader context, it is remarkable that small RNA-guided transcript slicing is repeatedly being used during evolution to initiate small RNA biogenesis in various pathways of plants, fungi, and animals (e.g., see Allen et al. 2005; Bagijn et al. 2012; Lee et al. 2012; Creasey et al. 2014).

## Materials and methods

### Genetics and transgenes

The germline GFP-Piwi cDNA transgene (inserted into attP40) (Markstein et al. 2008) was driven by a *nanos* promoter and contained a *vasa* 3′ UTR. Other transgenes used were the GFP-Piwi BAC rescue construct (Sienski et al. 2012), GFP-Nup107 (Katsani et al. 2008), MTD-Gal4, and shRNAi lines (all inserted into attP2) targeting *white*, GFP, *piwi*, *aub*, and *ago3* (Ni et al. 2011; Olivieri et al. 2012; Mohn et al. 2014). We established additional *aub*-sh lines in attP40 and attP33 (both second chromosome). All *aub*/*ago3* double GLKD experiments used *aub-*sh (attP40) and *ago3-*sh (attP2), except those that included the germline Piwi transgene (attP40), where *aub*-sh (attP33) was used with *ago3-*sh (attP2). The novel *piwi*, *aub,* and *ago3* alleles were isolated in a *white*^1118^ background isogenic for the second and third chromosomes using two independent gRNAs per gene (Supplemental Table S3). Frameshift alleles induced by independent gRNAs were analyzed in *trans* to avoid potential “off-target” effects. GFP-tagged Piwi and Aub BAC transgenes rescued the otherwise sterile *piwi* and *aub* transheterozygous combinations to full fertility.

### RT-qPCR

Primer sequences used for RT-qPCR are listed in Supplemental Table S3 (primer and gRNA sequences).

### Antibodies

The following mouse monoclonal antibodies against His-tagged fragments of Piwi, Aub, and Ago3 (amino acids 1–150) were generated by the Max F. Perutz Laboratories Monoclonal Antibody Facility: Piwi, 8C2-E4 (immunoprecipitation and immunofluorescence); Aub, 8A3-D7 (immunoprecipitation, immunofluorescence, and Western blotting); and Ago3, 5H12-G12 (immunoprecipitation and Western blotting) and 7B4-C2 (immunofluorescence). Rabbit anti-Piwi and anti-Armi were used for Western blotting. Rabbit antisera against His-tagged Rpb3 (Adelman et al. 2005) and against peptides from *rover* Gag, *mdg3* Gag, *I*-element ORF1, and *HeT-A* ORF (Supplemental Table S3) were raised by Eurogentec.

### RNA-FISH and immunohistochemistry

CAL Fluor Red 590-labeled Stellaris oligo probes were used to detect the various TE transcripts (Supplemental Table S2). For each probe set, 48 oligos were computationally filtered to map only to the cognate TEs. RNA-FISH was performed as in Mohn et al. (2014). Confocal stacks of egg chambers were acquired on a Zeiss LSM780 microscope. Confocal stacks were deconvoluted using Huygens Remote Manager version 3.0.2.

### Quantitative GFP-Piwi fluorescence imaging

Confocal stacks of DAPI and GFP-Piwi fluorescence of stage 7 egg chambers were acquired (Zeiss LSM780) with identical acquisition parameters for all samples. A script written in the Definians software suite performed the unbiased analysis of confocal stacks: Small somatic or large nurse cell nuclei were detected and classified using DAPI. Nuclear GFP-Piwi fluorescence intensity was measured in two dimensions at the Z-section showing the largest nuclear circumference for each nucleus for all somatic and nurse cell nuclei. For GFP-Piwi (BAC rescue construct), the ratio of nurse cell GFP signal and somatic follicle cell GFP signal was calculated, averaged for the same genotype, and set to 100% in controls. For germline GFP-Piwi, the average intensity of all nurse cell nuclei per egg chamber was calculated, and the genotype average was set to 100% in controls. Note that *nanos*-driven GFP-Piwi levels are much lower than for GFP-Piwi (BAC). Imaging of these required confocal settings that inadvertently also excited DAPI at a low level, leading to a background signal in the GFP channel; this was also observed for the *w*^1118^ strain without germline GFP-Piwi.

### TE consensus sequences

A FASTA sequence file with 122 TEs (Supplemental Document S1) was compiled by merging all *D. melanogaster* TE entries from Repbase (http://www.girinst.org/repbase) with those from BDGP_embl version 9.41. Pairwise BLAST (bl2seq) was used to identify identical or highly similar entries, from which only one (Berkeley *Drosophila* Genome Project [BDGP] nomenclature) was retained. Unique entries from either database were added. The *D. mel aurora* entry was replaced with the *D. sim ninja* entry, as the latter is more related to the insertions in the *D. melanogaster* reference genome.

### DNA-seq, ChIP-seq, RNA sequencing (RNA-seq), Piwi/Aub/Ago3 immunoprecipitations, and small RNA-seq

For DNA-seq, genomic DNA isolated from *white* GLKD ovaries was sheared to average 400 base pairs (bp), size-selected to 200–600 bp, cloned, and sequenced (paired-end 100 nt). RNA Pol II ChIPs were performed in biological triplicates with rabbit anti-Rbp3 according to Lee et al. (2006), processed to ChIP-seq libraries (Mohn et al. 2014), and sequenced (50-nt single end). PolyA plus and ribozero RNA-seq was performed as described in Mohn et al. (2014), and libraries were sequenced (paired end 50 nt). Cap-seq from Piwi-depleted ovaries was performed as in Gu et al. (2012).

Piwi, Aub, Ago3, and GFP-Piwi immunoprecipitations from ovary lysates were carried out as in Mohn et al. (2015) but with monoclonal antibodies against Piwi, Aub, and Ago3. Oxidation of small RNAs was as in Vagin et al. (2006).

### Computational analyses of deep sequencing data

DNA-seq, Pol II ChIP-seq, and RNA-seq reads were trimmed to bases 5–45, and paired-end RNA-seq reads were split into forward and reverse reads and mapped separately. After normalization using genome-unique mappers, Pol II ChIP-seq and RNA-seq reads were mapped to the TE consensus file with BWA, allowing up to three mismatches and two mappings per TE (LTR coverage). To calculate the TE-specific numerical read counts, we generated a TE consensus file in which all 40mer reads mapping to multiple TEs were masked, and reads falling into masked regions were omitted from analysis. TE promoter mapping reads of RNA Pol II ChIP libraries (biological triplicates for mutant genotypes and quadruplicates for control genotypes) were averaged (Supplemental Table S1, TE master table). Analysis of Cap-seq data was done as described (Mohn et al. 2014).

The approximate TE copy numbers in the used strains were calculated by dividing length-normalized TE matching read counts by average genic read counts.

TE promoter sequences were defined as follows: LTR sequences were used for LTR elements. The first 500 bp of LINEs and DNA elements were used. For *HeT-A*, *TART*, and *TAHRE* promoters, 500 bp centered on the Cap-seq peaks observed in Piwi depletion were used (Supplemental Document S1, TE promoter sequences).

For each genotype, Piwi (total), Aub, and Ago3 immunoprecipitation libraries were normalized relative to a total small RNA-seq library (normalized to 1 million sequenced miRNAs; parts per million miRNAs [ppm]) using the most asymmetrically distributed piRNA species. Similarly, for control ovaries, the ppm-normalized total Piwi immunoprecipitation library was used to normalize germline GFP-Piwi immunoprecipitation and soma-specific Piwi immunoprecipitation libraries (Piwi-IP from ovaries with Piwi depleted in germline) (Supplemental Fig. S1D; Mohn et al. 2015). The germline-specific Piwi libraries of all other genotypes were normalized relative to controls using the experimentally determined nuclear germline GFP-Piwi levels (Supplemental Fig. S2B,C). All shown piRNA profiles against TEs and in University of California at Santa Cruz (UCSC) browser screen shots were calculated with zero mismatches. Promoter-proximal antisense germline Piwi piRNAs were mapped to the 3-kb region downstream from the apparent CAP-seq peaks in Piwi depletion ovaries or otherwise to the first 3 kb of TEs in which CAP-seq peaks were not apparent (see Supplemental Table S1). All deep sequencing data sets used in this study are listed in Supplemental Table S4.

### Statistical analysis

TE promoter Pol II occupancy and germline Piwi piRNA levels from different genotypes were compared by one-tailed Wilcoxon matched pairs signed rank test. Levels of germline GFP-Piwi piRNA of different groups of TEs from control ovaries were tested by unpaired, one-tailed *t*-tests. The indicated significance values correspond to <0.05 (*), <0.01 (**), and <0.001 (***).

Illumina deep sequencing data sets have been deposited at NCBI Gene Expression Omnibus (GSE71775).

## Supplementary Material

Supplemental Material
