# Supplementary material for: piRNA-guided slicing of transposon transcripts enforces their transcriptional silencing via specifying the nuclear piRNA repertoire
Source: Genes Dev. 2015 Aug 15;29(16):1747–62. doi: 10.1101/gad.267252.115 (PMC4561483; doi:10.1101/gad.267252.115)
Supplement: Supplemental Material [file supp_29_16_1747__index.html]

Supplemental Material 

# piRNA-guided slicing of transposon transcripts enforces their transcriptional silencing via specifying the nuclear piRNA repertoire

## Supplemental Material

**Files in this Data Supplement:**

- Supp Text & Figures.pdf
