# Supplementary material for: piRNA-guided slicing of transposon transcripts enforces their transcriptional silencing via specifying the nuclear piRNA repertoire
Source: Genes Dev. 2015 Aug 15;29(16):1747–62. doi: 10.1101/gad.267252.115 (PMC4561483; doi:10.1101/gad.267252.115)

Supplemental Figures Legends and Supplemental Figures

for

piRNA-guided slicing of transposon transcripts enforces their transcriptional silencing via specifying the nuclear piRNA repertoire

Kirsten-André Senti[1], Daniel Jurczak[1], Ravi Sachidanandam[2] & Julius Brennecke[1]

Supplemental Figure S1: Efficient Piwi/Aub/Ago3 depletions by germline specific RNAi

(A) Color-inverted confocal images depict stage 7 egg chambers of indicated genotypes stained for Piwi, Aub or Ago3 (black; scale bar = 20μm).

(B) Shown are fold changes of *piwi*, *aub*, and *ago3* mRNA levels (relative to *rpL32*) in the indicated genotypes (n = 3; error bars indicate SD).

(C) Images show loading patterns of Ponceau stained membranes used for western blots shown in Fig. 1C.

(D) Scheme showing the two-step IP normalization strategy (details see methods) exemplified by piRNA profiles for the *blood* transposon. In step 1 a miRNA normalized total small RNA-seq library was used to obtain normalization factors for Ago3-, Aub and total-Piwi-IP libraries (v, w, x). In step 2 the normalized total-Piwi IP library was used to obtain normalization factors for germline Piwi-IP and soma Piwi-IP libraries (y, z).

Supplemental Figure S2: Impact of cytoplasmic ping-pong on germline Piwi-bound piRNA populations

(A) Color-inverted confocal images shows germline GFP-Piwi fluorescence (black) in stage 7 egg chambers of indicated genotype (scale bar = 20μm).

(B) Shown is the average nuclear GFP-Piwi fluorescence intensity (in % of control) of germline GFP-Piwi in ovaries of the indicated genotypes.

(C) Bar diagram shows the 5' Uridine-bias (1U; %) of germline Piwi-bound piRNAs isolated from ovaries of indicated genotypes.

(D) Histograms shows length profiles of germline Piwi-bound piRNAs isolated from ovaries of indicated genotypes.

(E) Scatterplot of the sum of normalized sense and antisense germline Piwi-bound piRNAs versus the sum of normalized sense and antisense Aub- and Ago3-bound piRNAs mapping to individual TEs.

(F) UCSC browser shots shows normalized Aub (orange) germline Piwi (green), and Ago3-bound (blue) piRNAs obtained from ovaries of indicated genotype and mapping to *tRNA-Glu-CTC* (all mappers), the *pasha* hairpin, or the *oskar* mRNA (unique mappers).

Supplemental Figure S3: Determination of TEs exhibiting strong piRNA pathway repression in the germline

(A) Shown are profiles of RNA Pol II occupancy (top) and Cap-seq reads (bottom) mapping to *blood* from control ovaries or from ovaries depleted for Piwi in the germline. The determined transcription start site (TSS) is indicated (red arrowheads).

(B) Flow chart illustrating the parameters that were used to classify TEs as piRNA pathway repressed. First, TE copy number was used to exclude ancient and probably inactive TEs (<2 genomic copies). Next, only TEs with a fold increase in Pol II occupancy over TE promoters larger than 1.5 in any genotype were considered. Finally, we restricted the analysis to TEs exhibiting steady state RNA levels > RPKM=2 under de-repressed conditions and exhibiting a minimal fold change in RNA levels in any GLKD > 3.

(C, D) Venn diagrams showing the number of TEs with Pol II occupancy changes of at least 1.5 fold in the respective genotypes. Note that 25 of 27 TEs shown in (D) are specifically deregulated in germline piRNA pathway depletions, but not in soma piRNA pathway depletions (our unpublished data). Exceptions are *blood* and *rover,* which are active and repressed in germline and somatic ovarian tissues alike.

Supplemental Figure S4. Silencing patterns of the *I*-element and *Burdock*, two Piwi/Aub/Ago3 TEs

(A, B) Shown are normalized profiles of piRNAs bound to germline Piwi (green), Aub (orange) or Ago3 (blue) mapping to the *I*-element (A) or *Burdock* (B) obtained from ovaries of indicated genotypes. Bar diagrams display the sum of respective piRNA populations (sense and antisense; in 1000 x ppm).

(C, D) Color-inverted confocal images of stage 7 egg chambers depicting *I*-element (C), *Burdock* (D) RNA-FISH (black) and *I*-element ORF1 protein immuno-localization (C) in ovaries of indicated genotype (scale bar = 20µm). Red asterisks mark single nuclei shown enlarged (scale bar = 2µm) with RNA-FISH (black) and GFP-Nup107 (green) outlines nuclei.

(E) Plots showing the ping-pong signatures and ping-pong Z-values (calculated from total small RNA-seq libraries) of all Piwi/Aub/Ago3 group TEs in the indicated genotypes.

Supplemental Figure S5. Silencing patterns of 3S18/*Bel*, a Piwi/Aub-dominant TE

(A) Plots showing the ping-pong signatures and ping-pong Z-values (calculated from total small RNA-seq libraries) of all Piwi/Aub dominant TEs in the indicated genotypes.

(B) Shown are normalized profiles of piRNAs bound to germline Piwi (green), Aub (orange), or Ago3 (blue) mapping to *3S18/Bel*. Bar diagrams indicate the sum of respective piRNA populations (sense and antisense; in 1000 x ppm).

(C) Color-inverted confocal images of stage 7 egg chambers depicting *3S18/Bel* RNA-FISH signals (black) in ovaries of indicated genotype (scale bar = 20μm). Enlarged captures (scale bar = 2μm) show RNA-FISH signal (black) and GFP-Nup107 marked nuclear envelope (green) for individual nurse cell nuclei (respective nuclei marked in left panel by red asterisks).

Supplemental Figure S6. Silencing patterns of *mdg3* and *Max*, two Piwi-dominant TEs

(A, B) Shown are normalized profiles of piRNAs bound to germline Piwi (green), Aub (orange) or Ago3 (blue) mapping to *mdg3* (A) or *Max* (B) obtained from ovaries of indicated genotypes. Bar diagrams indicate the sum of respective piRNA populations (sense and antisense; in 1000 x ppm).

(C, D) Color-inverted confocal images showing stage 5/6 egg chambers depicting *mdg3* (C) and showing stage 7 egg chambers with *Max* (D) RNA-FISH signals (black) as well as immuno-localization of *the mdg3* ORF protein (black) (C) in ovaries of indicated genotype (scale bar = 20μm). Enlarged captures (scale bar = 2μm) show RNA-FISH signal (black) and GFP-Nup107 marked nuclear envelope (green) for individual nurse cell nuclei (respective nuclei marked in left panel by red asterisks).

(E) Plots showing the ping-pong signatures and ping-pong Z-values (calculated from total small RNA-seq libraries) of all Piwi dominant group TEs in the indicated genotypes.

Supplemental Figure S7. Higher TE sequence divergence in the Piwi dominant group of TEs.

(A and B) Scatter plots of normalized total small RNAs from control ovaries mapped with 0 and 3 mismatches (x and y axis respectively) to those 70 TEs that make up more than 95% of all germline-Piwi piRNAs. Piwi dominant TEs are shown in blue, Piwi/Aub dominant TEs in red, Piwi/Aub/Ago3 TEs in green and TEs not deregulated by the germline-piRNA pathway in grey.

(C) Jitter plots with median bars show the ratio of total small RNAs mapping with 3 versus 0 mismatches. Labels as in (A, B).

(D and E) Scatter plots of normalized germline-Piwi piRNAs from control ovaries mapped with 0 and 3 mismatches (x and y axis respectively) to the same 70 TEs as in (A-C).

(F) Jitter plots with median bars of the ratio of 3 versus 0 mismatches as in (C, D).

Supplemental Figure S8. Characterization of novel *piwi*, *aub,* and *ago3* null alleles

(A) Shown are protein cartoons of Piwi, Aub, and Ago3 with PAZ and PIWI domains, CRISPR/Cas9 induced frame shifts (FS; black arrows), and short hairpin mediated RNA cleavage sites (blue arrows) at the indicated amino acid positions. Sequence changes between *white*^{1118} and the indicated homozygous mutant alleles of *piwi*, *aub* and *ago3* are indicated.

(B) 10μg total protein from ovary lysates of the indicated genotypes were resolved on 8% SDS-PAGE gels, transferred and probed for Piwi, Aub, or AGO3 and then re-probed for Armitage as a loading control (* indicates an unspecific band detected by the rabbit anti-Piwi antibody).

(C) Shown is the morphology of ovaries of the indicated genotype (scale bar: 500μm).

(**D**) Shown are confocal images of stage 7 egg chambers of indicated genotype stained for Piwi, Aub, or Ago3 (black; scale bar: 20μm).

(E) Shown are confocal images of stage 7 egg chambers of indicated genotype detecting germline GFP-Piwi fluorescence (black; scale bar: 20μm).

(F) Plot showing the average nuclear GFP-Piwi fluorescence intensities (in % of control) from a germline driven GFP-Piwi construct in the indicated genotypes. Error bars indicate the SD of at least 5 imaged egg chambers per genotype.

(**G**) Graphs showing fold changes of expression (relative to *rpL32*) of the indicated TEs by RT-qPCR (n=3; error bars indicate SD).

267252, Supplemental Figure 1, Senti et al.

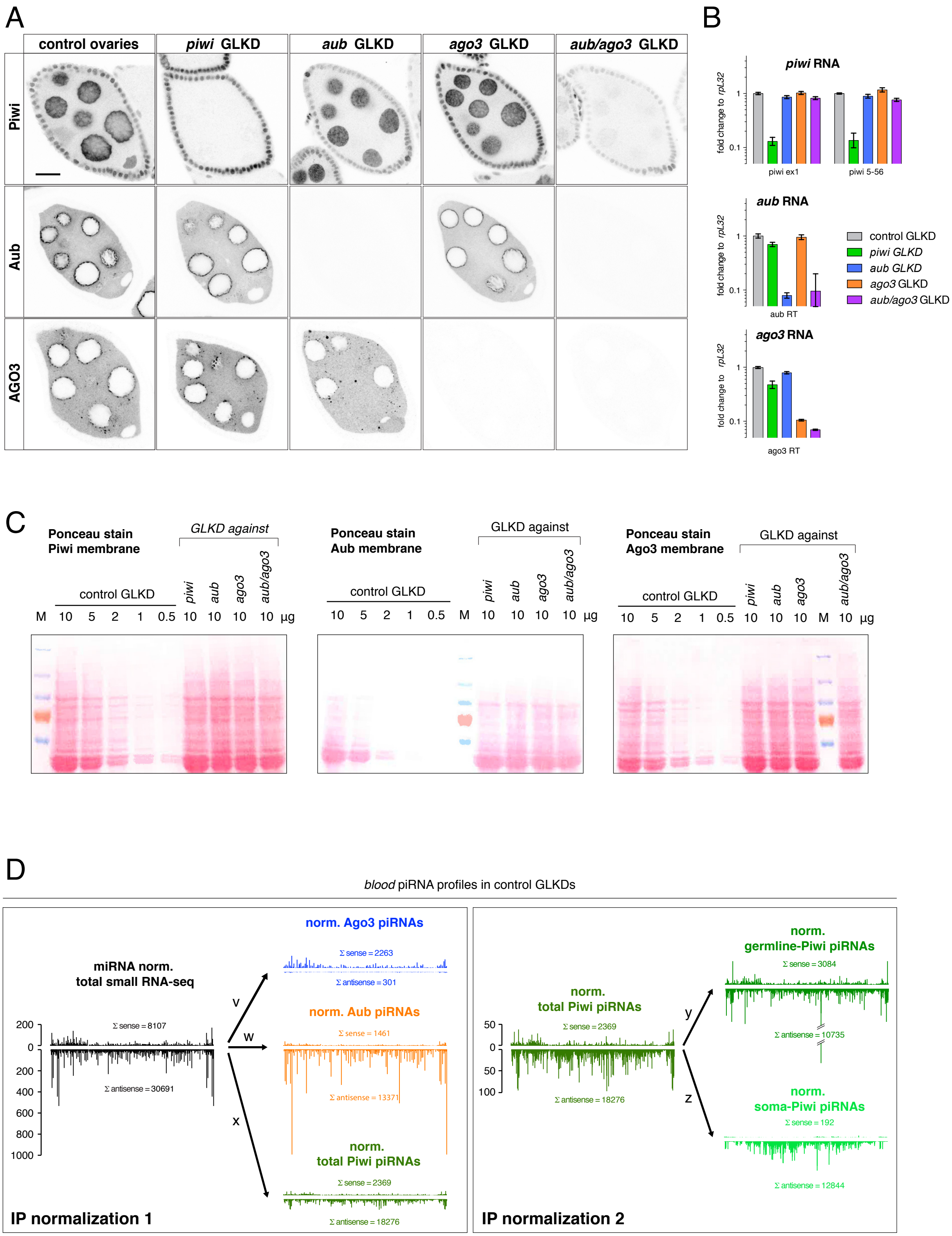

267252, Supplemental Figure 2, Senti et al.

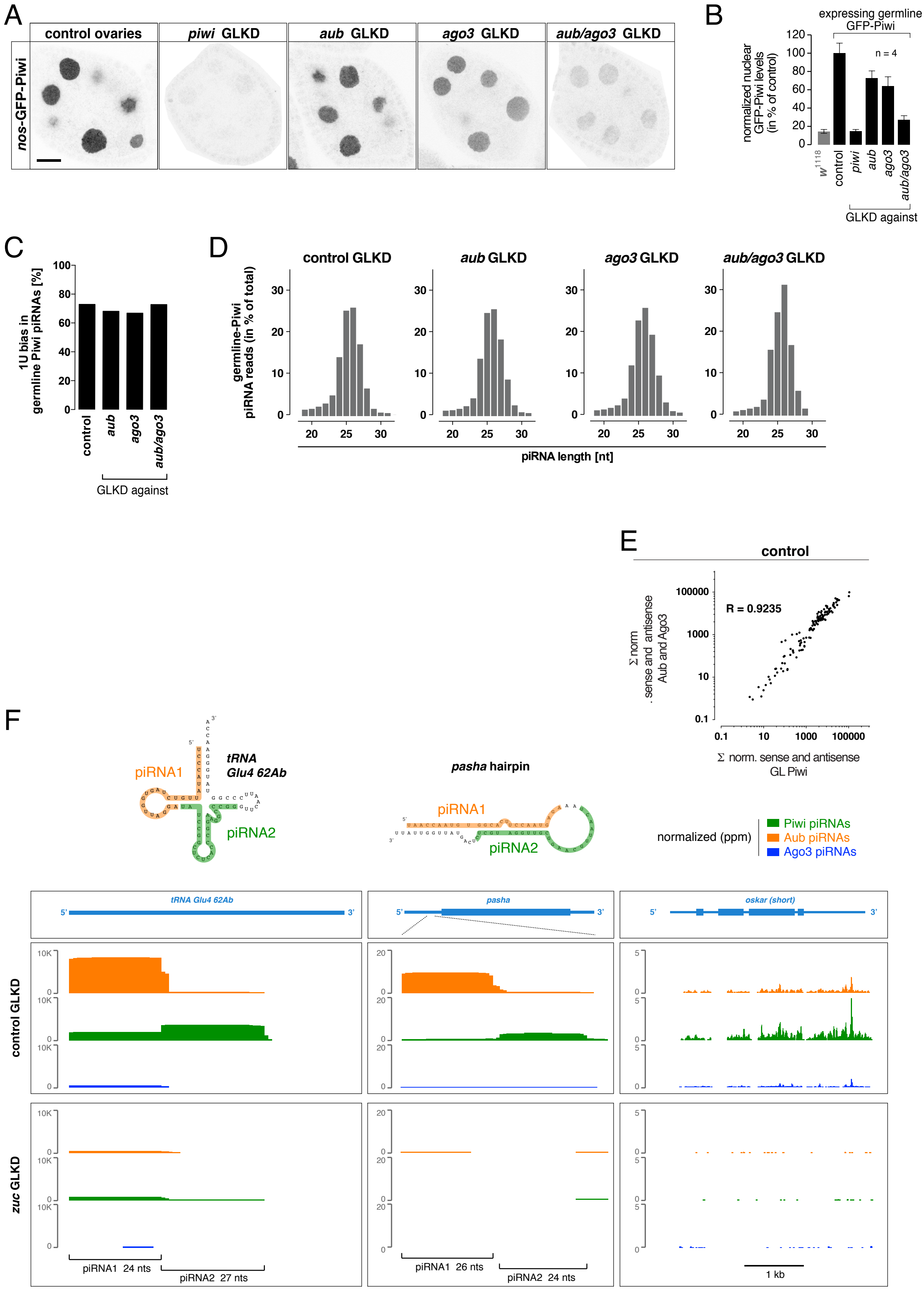

267252, Supplemental Figure 3, Senti et al.

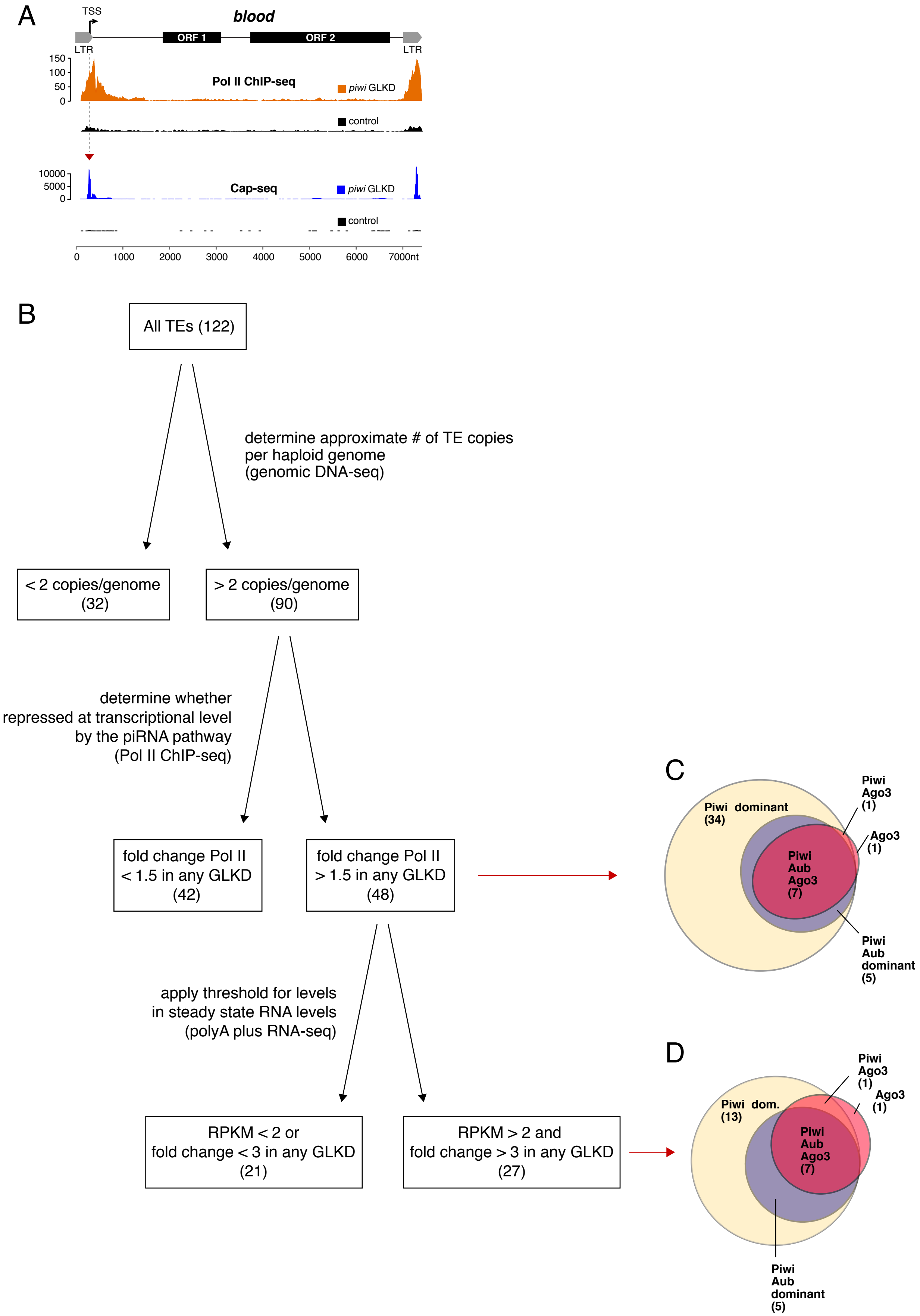

267252, Supplemental Figure 4, Senti et al.

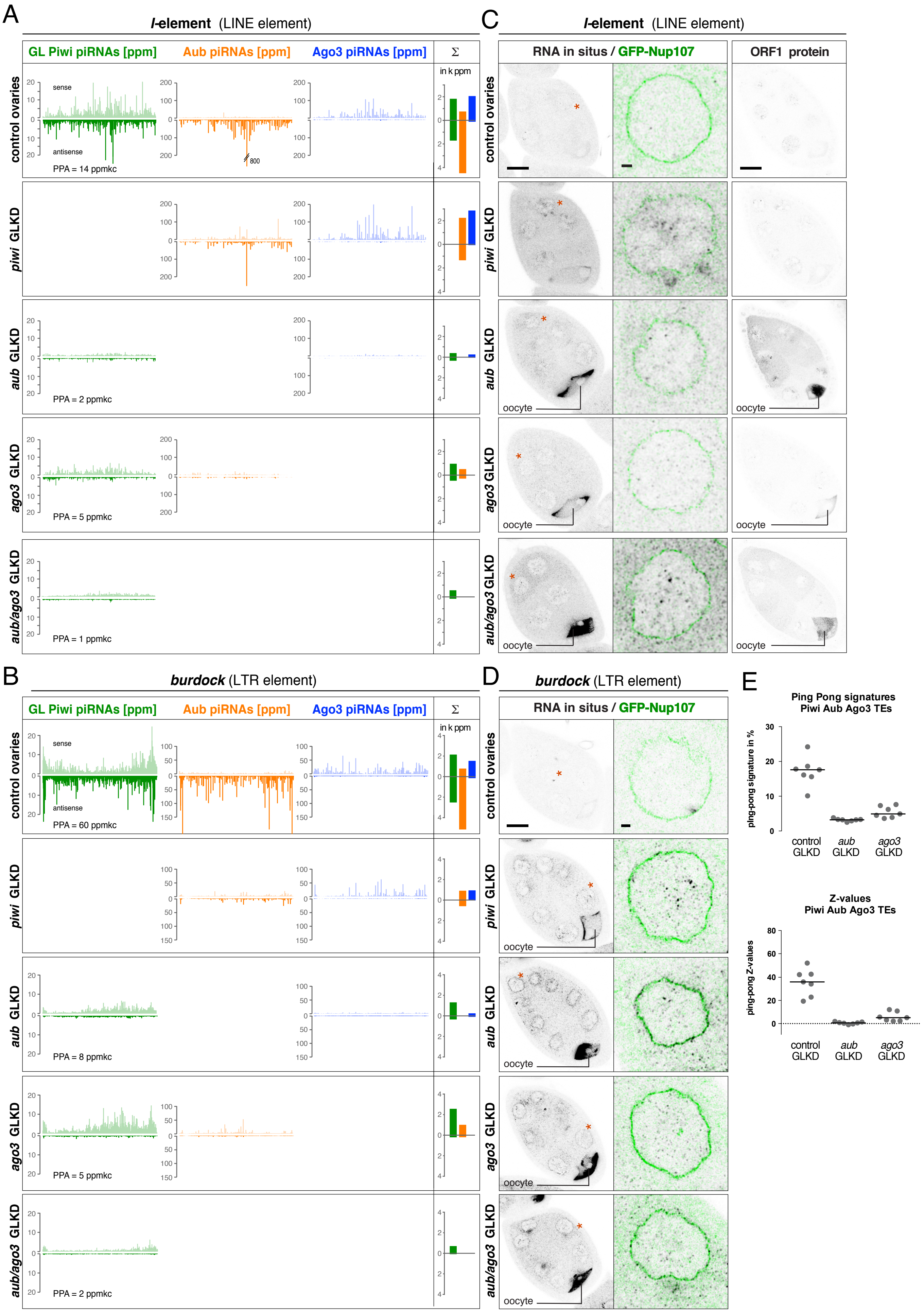

267252, Supplemental Figure 5, Senti et al.

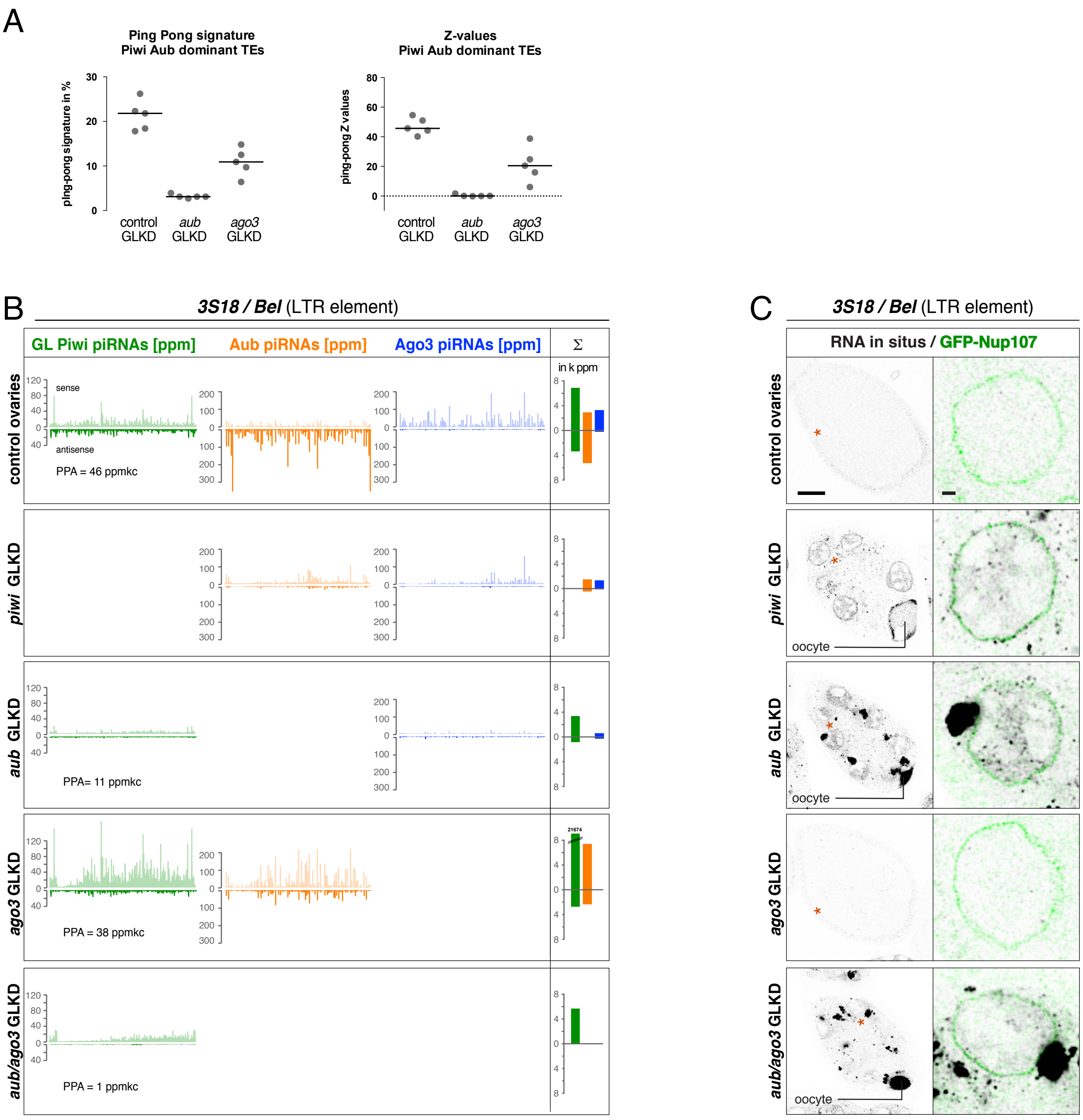

267252, Supplemental Figure 6, Senti et al.

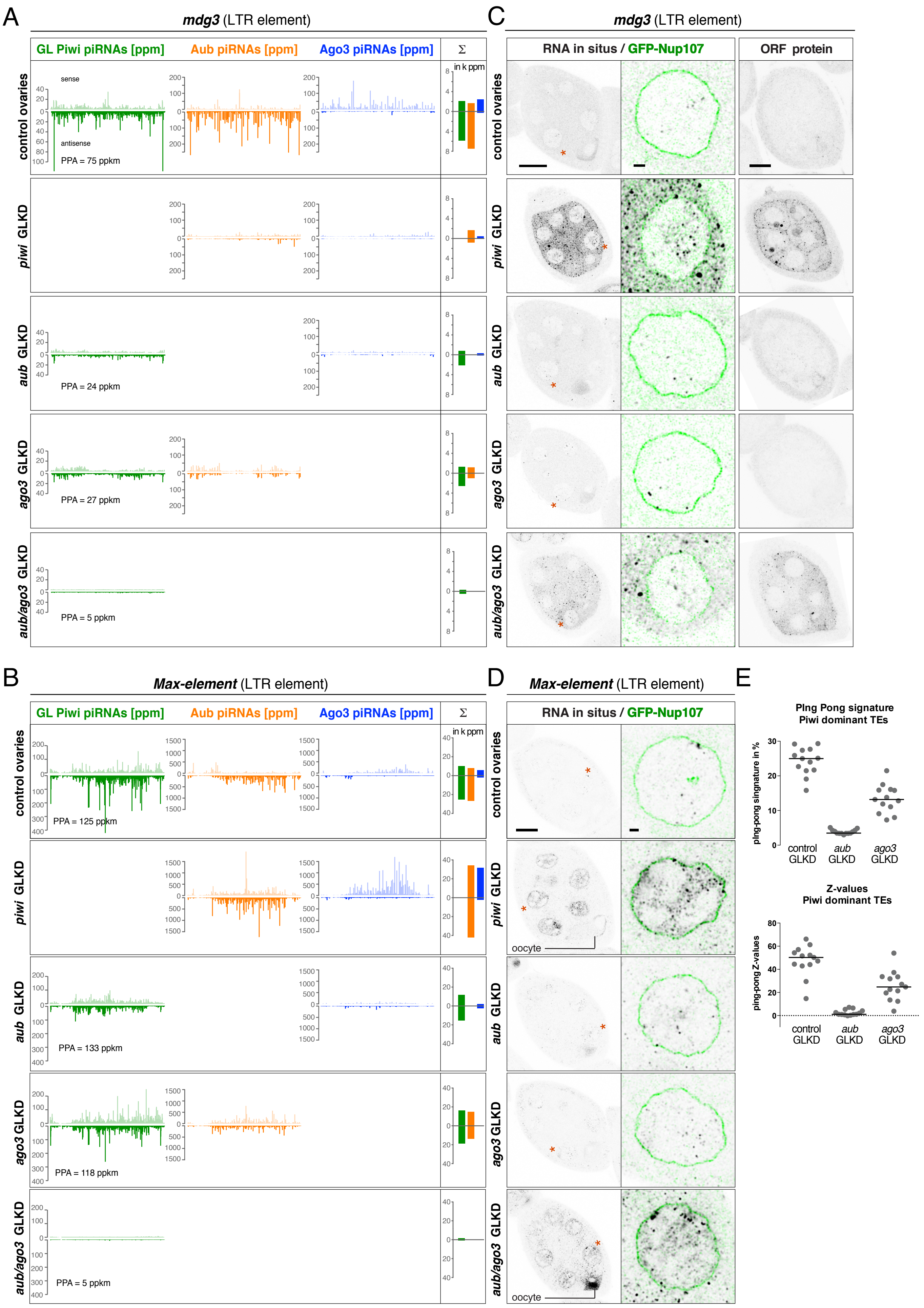

267252, Supplemental Figure 7, Senti et al.

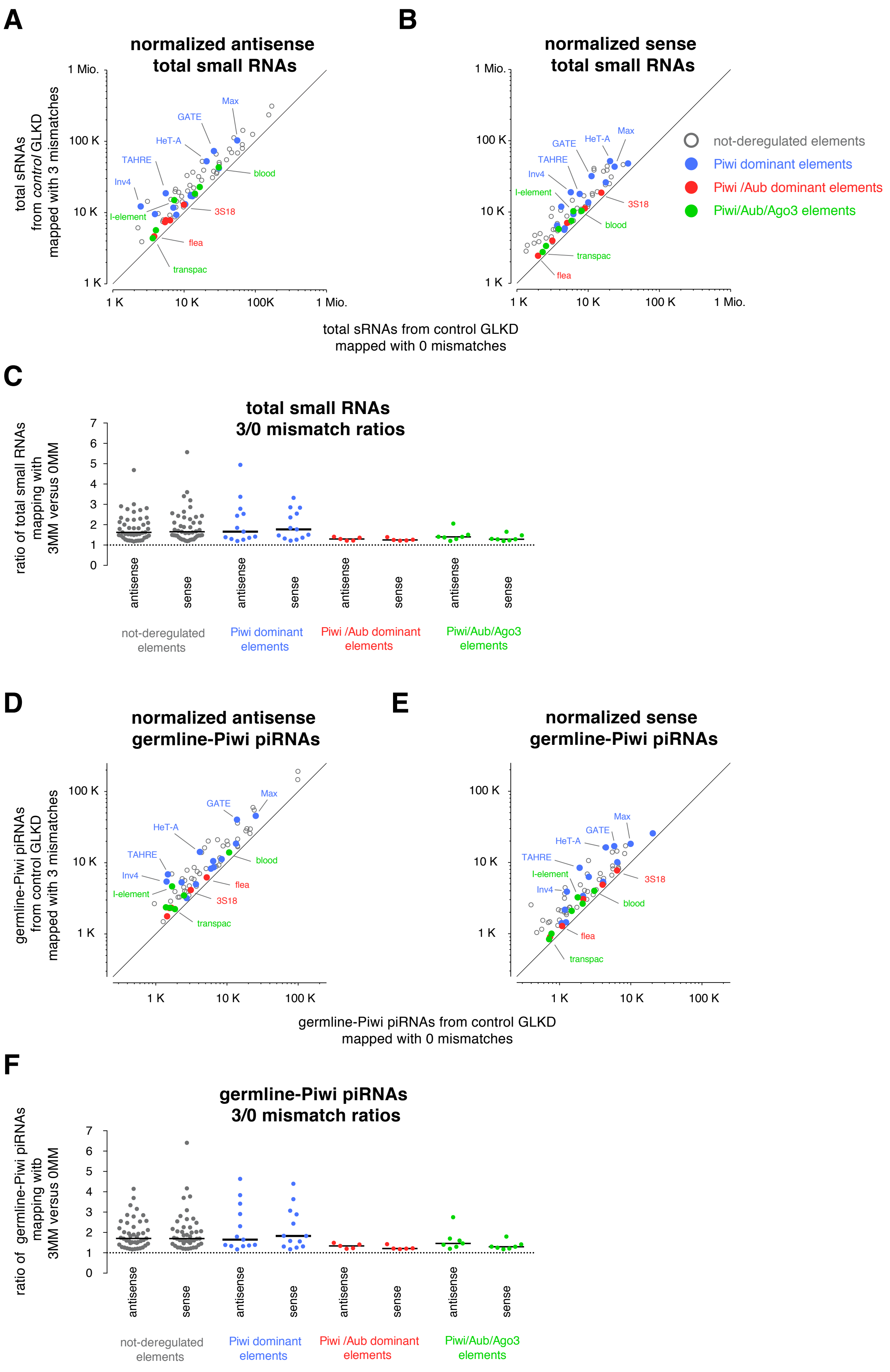

267252, Supplemental Figure 8, Senti et al.

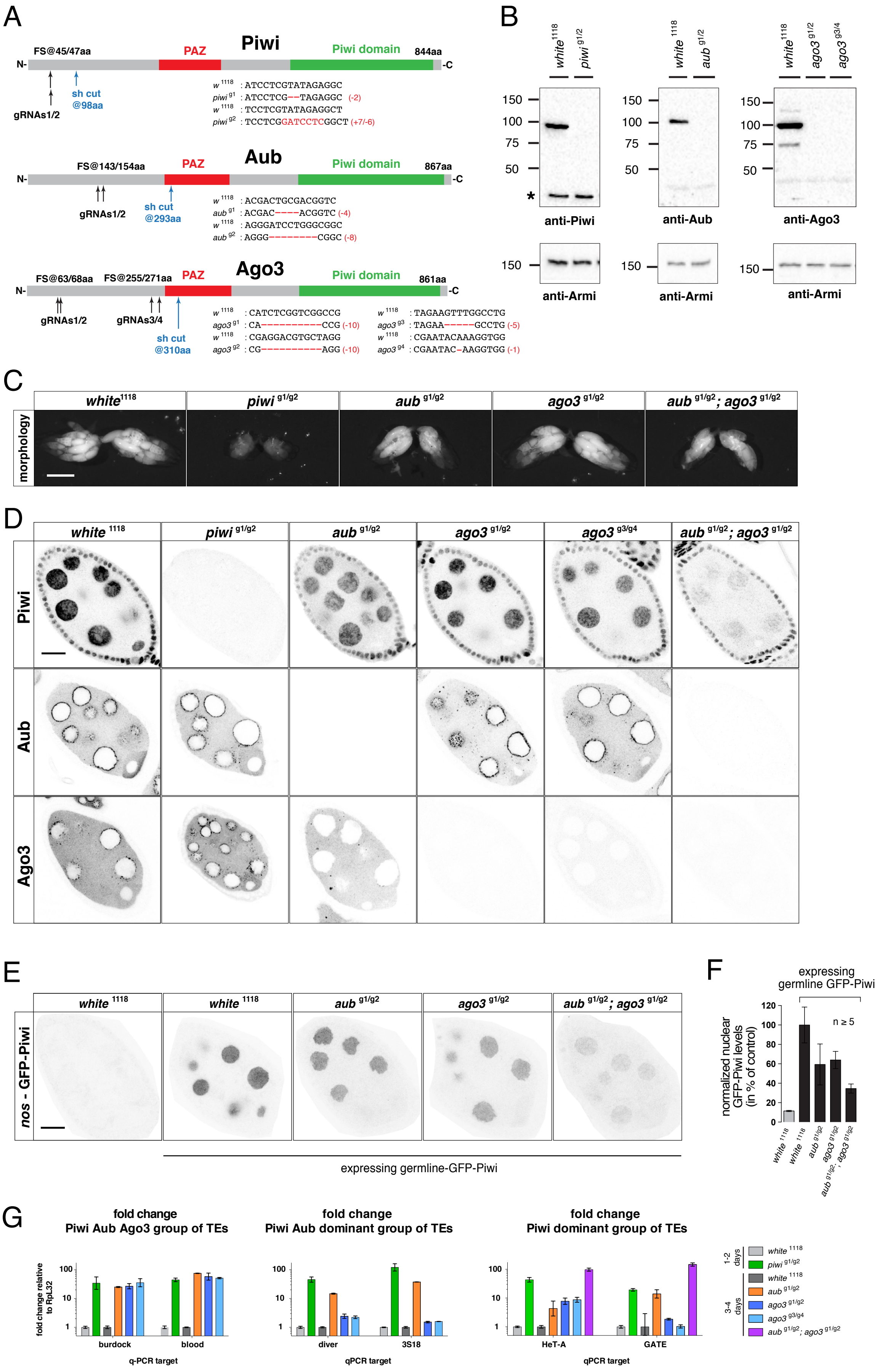

Supplement: Supplemental Material [file supp_gad.267252.115_SuppText_Figures.pdf]
